# Asynchronous Double Schiff Base Formation of Pyrazole Porous Polymers for Selective Pd Recovery

**DOI:** 10.1002/advs.202001676

**Published:** 2021-03-02

**Authors:** Mousumi Garai, Manmatha Mahato, Yeongran Hong, Vepa Rozyyev, Uiseok Jeong, Zakir Ullah, Cafer T. Yavuz

**Affiliations:** ^1^ Department of Chemical and Biomolecular Engineering Korea Advanced Institute of Science and Technology (KAIST) 291 Daehak‐ro, Yuseong‐gu Daejeon 34141 Korea; ^2^ Graduate School of Energy, Environment, Water and Sustainability (EEWS) KAIST Daejeon 34141 Korea; ^3^ Department of Chemistry KAIST Daejeon 34141 Korea; ^4^ Advanced Membranes and Porous Materials Center (AMPM), Physical Sciences and Engineering (PSE) King Abdullah University of Science and Technology (KAUST) Thuwal 23955–6900 Saudi Arabia

**Keywords:** electronic waste, kinetic products, precious metal capture, urban mining, water treatment

## Abstract

Pyrazole‐linked covalent organic polymer is synthesized using an asynchronous double Schiff base from readily available monomers. The one‐pot reaction features no metals as a building block or reagent, hence facilitating the structural purity and industrial scalability of the design. Through a single‐crystal study on a model compound, the double Schiff base formation is found to follow *syn* addition, a kinetically favored product, suggesting that reactivity of the amine and carbonyls dictate the order and geometry of the framework building. The highly porous pyrazole polymer COP‐214 is chemically resistant in reactive conditions for over two weeks and thermally stable up to 425 °C in air. COP‐214 shows well‐pronounced gas capture and selectivities, and a high CO_2_/N_2_ selectivity of 102. The strongly coordinating pyrazole sites show rapid uptake and quantitative selectivity of Pd (II) over several coordinating metals (especially Pt (II)) at all pH points that are tested, a remarkably rare feature that is best explained by detailed analysis as the size‐selective strong coordination of Pd onto pyrazoles. Density functional theory (DFT) calculations show energetically favorable Pd binding between the metal and N‐sites of COP‐214. The polymer is reusable multiple times without loss of activity, providing great incentives for an industrial prospect.

## Graphical abstract

One step, consequential imine linking produced the first pyrazole network polymers with an unusual selectivity for the *syn* addition. Proven by single‐crystal studies, a kinetic controlled product provides tailor‐made pockets for rapid, pH independent, reversible Pd binding and an exceptional selectivity over other strongly coordinating precious metals like platinum.

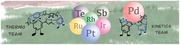

## 1 Introduction

The design, assembly, and utilization of advanced porous materials with precise architectures and prominent features^[^ ^1^, ^2^, ^3^, ^4^ ^]^ have revolutionized emerging applications in energy and the environment with the development of structures ranging from inorganic silica,^[^ ^5^ ^]^ activated carbon,^[^ ^6^ ^]^ zeolites,^[^ ^7^ ^]^ and metal–organic frameworks^[^ ^8^ ^]^ to covalent organic networks.^[^ ^9^, ^10^ ^]^ Similarly, an evolving class of porous organic polymers with potential applications in gas capture, separation, water treatment, metal capture, and catalysis have become a major research field in materials chemistry;^[^ ^11^, ^12^, ^13^, ^14^, ^15^ ^]^ and the use of rigid building units with multiple covalent connectivities led to tunable surface areas, permanent porosity, and robust nature. The diversity of the building blocks, tied with the extensive availability of linkers allowed the development of new porous materials, such as polymers of intrinsic microporosity,^[^ ^16^ ^]^ azo‐linked polymers^[^ ^17^, ^18^ ^]^ Schiff base networks,^[^ ^19^, ^20^ ^]^ nanoporous benzoxazole networks,^[^ ^21^ ^]^ porous aromatic frameworks,^[^ ^22^ ^]^ covalent organic frameworks (COFs),^[^ ^23^ ^]^ and covalent organic polymers (COPs).^[^ ^24^ ^]^

Porous materials could make a great impact in the area of precious‐metal recovery from industrial waste.^[^ ^25^, ^26^ ^]^ Stability in strongly acidic media and cost cutting by multiple‐time reuse promises great incentives for the electronics and car industries to reclaim Pt‐group metals. Despite the urgency, the highly selective recovery of individual precious metals (such as Pd from others) was not properly addressed using porous materials. This is predominantly because the chemistry to build robust network structures does not always feature highly coordinating nitrogen groups. For example, porous organic polymers were mostly made by several popular chemical reactions, such as the Suzuki cross‐coupling,^[^ ^27^ ^]^ Sonogashira–Hagihara,^[^ ^28^, ^29^ ^]^ oxidative coupling,^[^ ^30^, ^31^ ^]^ Schiff base,^[^ ^32^, ^33^, ^34^ ^]^ and Friedel–Crafts arylation.^[^ ^35^ ^]^ Among them, a promising strategy to prepare high heteroatom‐containing porous structures is by using Schiff base, which forms dynamic imine bond chemistry between reactive carbonyl and amines. The main drawback of imine‐linked polymers is the physiochemical stability under acidic/basic conditions and high temperature. Banerjee and co‐workers have reported restricted tautomerism phenomena and locking of the keto‐enamine form of COF to enhance acid/base stability.^[^ ^36^ ^]^ Some of these challenges have been fixed by locking with intralayer hydrogen bonding interactions of COFs by Chen et al. to improve physical properties.^[^ ^37^ ^]^ Recently, Coskun and co‐workers showed an excellent strategy to synthesize conjugated microporous polymers by a Schiff base reaction followed by an acid‐catalyzed in situ cyclization reaction to improve stabilities.^[^ ^38^ ^]^ But these strategies had limited scope to improve stabilities. In addition, problems of metal‐catalyzed polymerization are metal impurities, pore blocking, and not being cost effective.

Herein, we present a facile, one‐pot, metal‐free synthetic strategy to synthesize the first example of pyrazole‐connected porous COPs using a double Schiff base, which connect the aromatic bis‐hydrazine with the tris‐acetylacetonate linker to achieve highly stable porous frameworks. This approach enabled us to form the kinetic product,^[^ ^39^, ^40^ ^]^ which was confirmed by the model system through single‐crystal analysis. Interestingly, *syn* addition was favored over *anti*. The synthesized pyrazole polymer, COP‐214 exhibited microporosity and a strongly chelating coordination site,^[^ ^41^, ^42^ ^]^ prompting us to test for precious metal recovery. Surprisingly, COP‐214 shows an exceptional selectivity for Pd(II) ions over other metal ions such as Pt, Ru, Rh, Sb, Ir, V, Cr, Mn, Fe, Co, Ni, Cu, Cd, Tl, Pb, and U with a significantly enhanced adsorption rate. Selective recovery of a precious metal from e‐waste without any subsequent waste treatment process shows great promise for the urban mining of precious metals from industrial waste. In order to gain more insights into the reaction mechanism, we carried out DFT calculations for the binding behavior of Pd and Pt in COP‐214, and the results confirm the preference of Pd binding with the N‐sites of COP‐214, instead of Pt.

## 2 Results and Discussion

### 2.1 Synthesis and Characterization of Porous Pyrazole Polymers

**Figure 1:**
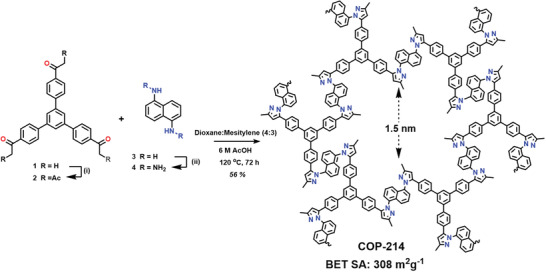
Synthesis of COP‐214 from commercially available monomers in several steps using an asynchronous double Schiff base with a surface area of 308 m^2^ g^−1^ and an average pore width of ≈1.5 nm. Reaction conditions: i) Ac_2_O, BF_3_.AcOH, Et_3_N, MeOH, 50 °C, 12 h. ii) Concentrated HCl, NaNO_2_, −10 °C, 1.5 h, then urea 1 h, SnCl_2_, −15 °C overnight to 0 °C and workup with NaOH (20% w/w) (The total yield from **2** and **4** to COP‐214 is 56%. See Supporting Information for synthetic details). For clarity, the extended COP‐214 structure is not shown.

To achieve the first example of a porous pyrazole polymer with a high loading of heteroatoms, we chose acetylacetonate tris‐phenyl benzene (**2**) and 1,5‐naphthalene di‐hydrazine (**4**) monomers units and synthesized them following reported procedures.^[^ ^43^, ^44^, ^45^, ^46^ ^]^ The COP‐214 was, therefore, constructed by performing a sealed ampoule reaction between 2 and 4 through an asynchronous, double Schiff base at 120 °C with 6 m AcOH for 72 h (**Figure** **1**). After a judicious solvent screening, we successfully achieved a porous polymer using 1,4‐dioxane:mesitylene (4:3) solvent combination (see Table S5, Supporting Information, for the experimental details). In addition to the conventional heating method, the porous pyrazole polymer was also prepared using microwaves at 120 °C for 2 h in a dioxane:mesitylene (4:3) solvent system (COP‐214‐MW), mainly to enable a scale‐up for industrial applications. The precipitates were collected and washed repeatedly with dioxane, methanol, and acetone. The solids were dried under vacuum at 90 °C overnight.

**Figure 2:**
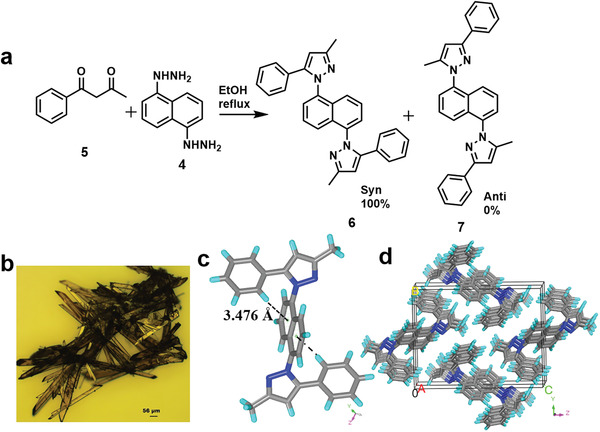
Model compound proves kinetic product formation: a) Synthesis of model compound **6** from **4** and **5** using double Schiff base under reflux conditions. b) Optical images of grown crystals; brown color, thin rod shape, with scale bar, ≈56 um. c) Structure of model compound **6** showing weak C—H⋯*π* interaction (3.476 Å), distance between centroid of central phenyl ring and edge site hydrogen of phenyl unit after the diffraction of single crystals. See Supporting Information for data and methods on the single‐crystal X‐ray diffraction (XRD) analysis. d) Unit cell packing of **6** along *YZ* plane.

The asynchronous, double Schiff base makes it important to investigate the sequence of imine formation on the product. For *syn* addition, the more reactive N on the phenyl hydrazine (—NH_2_) needs to attack the more reactive carbonyl, the outermost on the acetylacetone (acac) unit. Since *syn* addition product formation is sterically challenged, we believe it is kinetically favored (**Figure** **2**a). On the other hand, an *anti*‐addition adduct is more thermodynamic stable, although it requires the more reactive hydrazine nitrogen to react with the more *π*‐delocalized, resonance stabilized *β*–keto enol unit.

To verify whether *syn* or *anti* addition is prevalent in the COP‐214 formation, we have designed and synthesized a model compound starting from a 1‐phenylbutane‐1,3‐dione (**5**) and naphthalene di‐hydrazine (**4**) under reflux conditions in ethanol. In order to get further structural insights into the model monomer, a series of spectroscopic techniques (^1^H NMR, ^13^C NMR, 2D ^1^H nuclear Overhauser effect (NOESY), 1D ^1^H nuclear Overhauser effect (NOE), and LC‐MASS) were carried out. NOESY NMR spectra show correlation signals between aromatic moieties due to close proximity, but no cross peak was observed between the methyl group and aromatic units (Figure S7a, Supporting Information). Additionally, data from the 1D NOE experiment shows that a strong peak at 6.64 ppm due to the —CH of acetylacetonate segment after specific irradiation of the methyl group as shown in Figure S7b, Supporting Information. Since aromatic signals are not affected, they are expected to be further away from the methyl group, confirming the NOESY readings. The adduct **6** exhibited a peak at m/z 441.2154 in the LC‐MS spectrum, indicating pyrazole formation in Figure S8, Supporting Information. To analyze the exact geometry, the model compound (**6**) was crystallized from a mixture of acetonitrile and DMF (ratio 2:1) (Figure 2a). After 7 days, brown‐colored single crystals of 6 were obtained and analyzed by single‐crystal XRD (Figure 2b). Single crystals of 6 revealed that it is crystallized in a monoclinic P2_1_/c space group. The asymmetric unit comprises one unit of **6**. The model compound **6** is stabilized by CH⋯*π* interactions (3.476 Å) of naphthalene moieties as shown in Figure 2c. Each molecule of 6 stacks via CH⋯*π* interactions (3.279 Å) along the *X*‐axis as shown in Figure S21, Supporting Information. The pyrazole units of 6 exhibit *anti* orientation (Figure S22b, Supporting Information). The unit of 6 is propagated through CH⋯N interactions along the *YZ* plane (Figure 2d). The model compound clearly shows a quantitative conversion to the kinetically favored *syn* product (Figure 2), therefore we believe that COP‐214 will predominantly feature sterically challenged *syn* additions. We anticipate that the framework structure will be more porous as the interlayer packing of the 2D planes will be less feasible.

**Figure 3:**
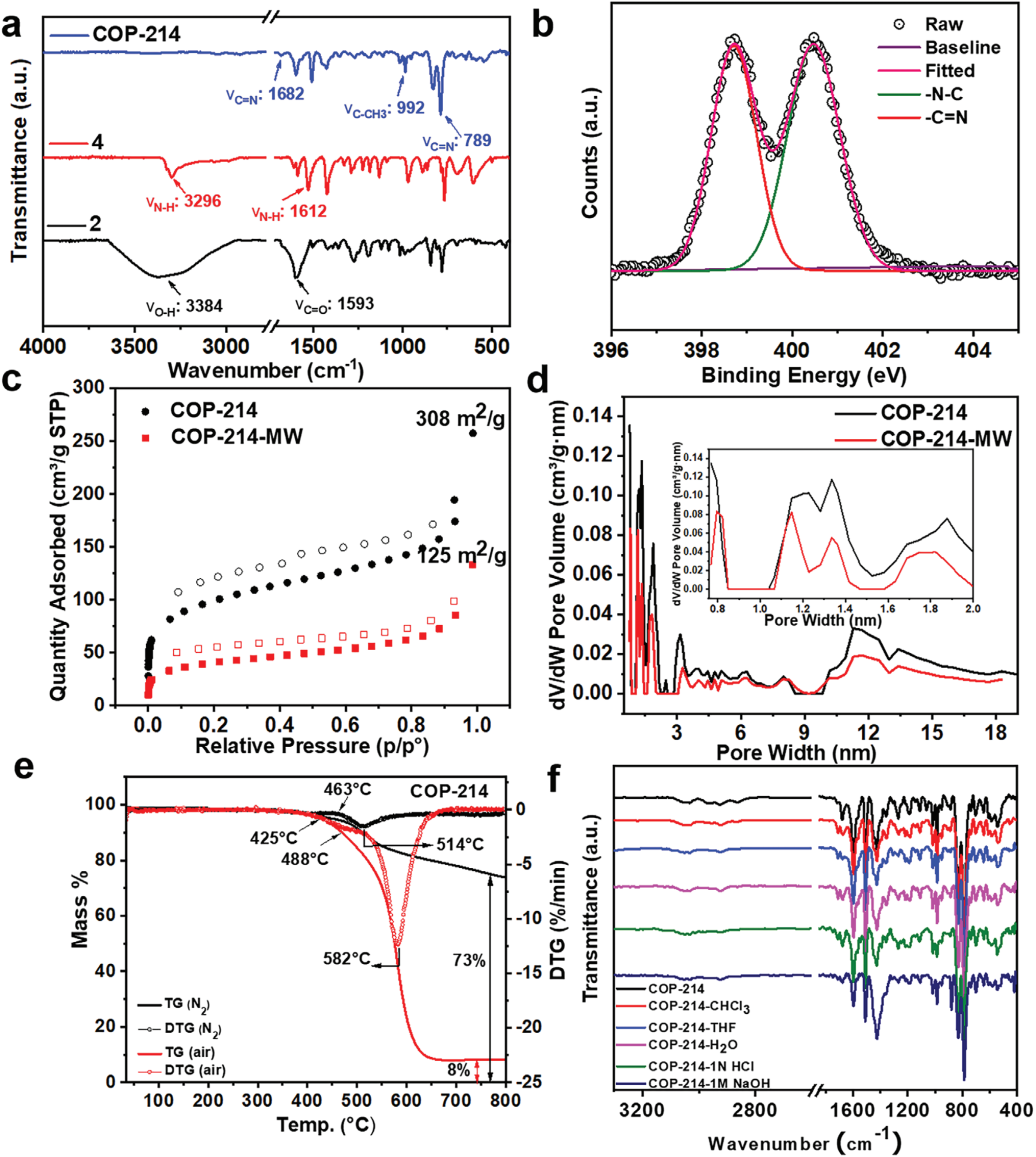
a) Comparisons of the FTIR spectra of COP‐214 and starting monomers (**2** and **4**) show the absence of starting materials in the product and the characteristic peaks corresponding to the formation of the pyrazole unit of the polymer superstructure. b) XPS analyses of N1s indicating the formation of pyrazoles in COP‐214. The spectrum of the N1s peak is deconvoluted using Origin 9.60 software. The functional groups corresponding to the deconvoluted peaks are shown. c) Ar physisorption isotherms of COP‐214 at 87 K, the type II reversible isotherms showing permanent micropores with surface area of COP‐214 308 m^2^ g^−1^ and COP‐214‐MW 125 m^2^ g^−1^ respectively, where filled and empty symbols represent adsorption and desorption, respectively. d) Pore‐size distribution of COP‐214 according to the non‐local density functional theory (NLDFT), which appears in the pore range of 0.8 to 20 nm suggesting a wide range of micro‐ and mesopore structures. The inset graph is the corresponding pore‐size distribution in the range of the 0.8 to 2 nm micropore region. e) Thermogravimetric analysis (TGA) of COP‐214 under nitrogen atmosphere (463 °C) and air (425 °C) showing high thermal stability. f) Stability of COP‐214 in various solvents, as indicated by their FTIR spectra after two weeks of treatment. All characteristic FTIR peaks remain after treatment, with no extra peaks corresponding to the collapse of the polymer structure.

The formation of the core pyrazole unit in COP‐214 was verified by FTIR, solid‐state NMR, and XPS spectra. The FTIR spectra of the COP‐214 depicts the absence of —NH stretching around 3296 cm^−1^, O—H stretching around 3384 cm^−1^, and —C=O stretching frequency at 1593 cm^−1^. A closer examination highlighted some unique pyrazole peaks at 1682 cm^−1^ (C=N stretching), 789 cm^−1^ (C=N out of plane) along with other characterized signals around 3046 and 2947 cm^−1^ (aromatic C‐H stretching) (not clearly visible due to the stacking of three spectra), 1598 cm^−1^ (aromatic C=C stretching), 830 cm^−1^ (aromatic C‐H out of plane), 1429 cm^−1^ (CH_3_ stretching), 1017 cm^−1^ (CH_3_ rocking), and 992 cm^−1^ (C—CH_3_ bending) (**Figure** **3**a). The molecular connectivity of COP‐214 was elucidated by solid‐state CP/MAS ^13^C NMR spectroscopy. The CP/MAS ^13^C NMR spectra of COP‐214 were found to be in perfect agreement with that of the model structure (Figure S9, Supporting Information). In particular, the carbon atom of the —C=CH and —CH_3_ of the acetylacetonate unit which resonated at 13.7 and 106.5 ppm, shows evidence of the presence of an acetylacetonate fragment. Additional carbon peaks of aromatic moieties located at 149.1, 145.6, 137.1, 131.1, 129.9, 128.8, 127.1, 126.8, and 124.1 ppm in the model compound were also observed in COP‐214. XPS data was also in line with the proposed structure, where N1s spectra revealed the existence of the pyrazole nitrogens at 398.89 (—C=N, imine) and 400.65 (—N—C) eV, respectively (Figure 3b).^[^ ^47^ ^]^ The observed two signals show equal peak area, indicating the presence of two electronically nonequivalent nitrogens in the pyrazole unit.

To evaluate the textural properties and porosity of the pyrazole polymers (COP‐214 and COP‐214‐MW) Ar adsorption–desorption isotherms were studied at 87 K showing typical type II reversible isotherms and the initial Ar uptake at low pressures displayed the characteristics of permanent micropores (Figure 3c). The specific surface areas of COP‐214 and 214‐MW were determined by the Brunauer–Emmett–Teller (BET) method and found to be 308 and 125 m^2^ g^−1^, respectively, with a wide range of micro and mesopores. The pore‐size distribution was calculated using NLDFT with infinite slit pore approximation. The pyrazole polymers exhibited a narrow pore‐size distribution in the microporous region. COP‐214 and COP‐214‐MW had similar features containing four major pore sizes below 2 nm, viz. 0.77, 1.15, 1.35, and 1.81 nm, consistent with the microporous nature of polymers (Figure 3d). The micropore volumes were determined by the t‐plot method and were found to be 0.042 and 0.016 cm^3^ g^−1^ for COP‐214 and COP‐214‐MW, respectively. To get more insight into the porous nature of the pyrazole polymers, the N_2_, CO_2_, CH_4_, and H_2_ adsorption isotherms of COP‐214 were recorded (see Figures S12 and S13, Supporting Information, for details about gas sorption and selectivity studies of COP‐214). CO_2_‐philic COP‐214 shows high CO_2_/N_2_ selectivity of 102 at 273 K (Figure S13a, Supporting Information) and the Q_st_ value of COP‐214 for CO_2_ at three temperature points (273, 298, and 323 K) was found to be in the range from 32.9 to 33.1 kJ mol^−1^ (Figure S13c, Supporting Information). The morphology of the synthesized material was studied by scanning electron microscopy and transmission electron microscopy (TEM) analysis and appears to have similar microporous polymers (Figure S10, Supporting Information).

Thermogravimetric analysis (TGA) confirmed the stability of COP‐214 up to 463 °C in N_2_ atmosphere and 425 °C in air (Figure 3e). TGA was performed under N_2_ and air flow to evaluate the thermostability of COP‐214 and to confirm the absence of trapped solvents inside the pores. Prior to analysis, the polymer was activated at 100 °C under vacuum for 12 h. COP‐214 exhibited no noticeable weight loss during the solvent evaporation bands (<300 °C). In the derivative thermogravimetry (DTG) curves, two degradation humps were observed under air at 425 and 488 °C for the decomposition of less stable functional moieties and the main framework, respectively. Whereas, under an inert atmosphere (N_2_ environment), the degradation of COP‐214 starts from 463 °C and continues uninterrupted. This implies that the thermal stability of COP‐214 is higher under inert conditions. It was also observed from the TGA that COP‐214 retained 73% of its initial mass after heating up to 800 °C in an N_2_ atmosphere. On the other hand, only 8% of the initial mass remained when COP‐214 was heated up to 800 °C under air due to oxidative degradation of the organic polymers at elevated temperatures. DTG plots show that the maximum rate of degradation occurred at 514 °C in air and at 582 °C in an N_2_ atmosphere. The results demonstrate the robust nature of the new polymers, which is expected since heterocycles are known to be thermally stable. For example, our recent report on benzoxazole polymers showed exceptional thermal stability.^[^ ^48^, ^49^ ^]^

Acid–base stability is a crucial part in a wide range of applications, especially in precious metal capture. The COP‐214 was immersed in various solvents (H_2_O, THF, CHCl_3_), and in harsh conditions like 1 n HCl or 1 m NaOH for 2 weeks. To investigate the stability of COP‐214 in different solvents, 50 mg of COP‐214 was directly submerged in 10 mL of varying solvents (H_2_O, THF, CHCl_3_), and harsh conditions like 1 n HCl or 1 m NaOH for 2 weeks. COP‐214 remained stable under these conditions. All of the characteristic FTIR peaks remained the same after treatment, and no additional peaks corresponding to the decomposition of materials were observed. The retention of peaks in the IR spectra confirmed resistance to acid and base digestion. The post‐treatment FTIR indicated no loss of structural integrity as shown in Figure 3f.

### 2.2 Precious Metal Selectivity of COP‐214

Highly selective sorbents are always in high demand, particularly in the field of precious metal recovery. The classical strategy for the synthesis of such sorbents is based on the introduction of nitrogen and sulfur‐containing functional groups to the polymer backbone. Due to the strong chelation potential of N‐sites, COP‐214 was studied for selective metal recovery from a mixture of precious metal solutions of electronic waste. The adsorption performances of COP‐214 in solutions containing a mixture of the metal ions of Pd, Pt, Ru, Rh, Sb, Ir, V, Cr, Mn, Fe, Co, Ni, Cu, Cd, Tl, Pb, and U were investigated.

**Figure 4:**
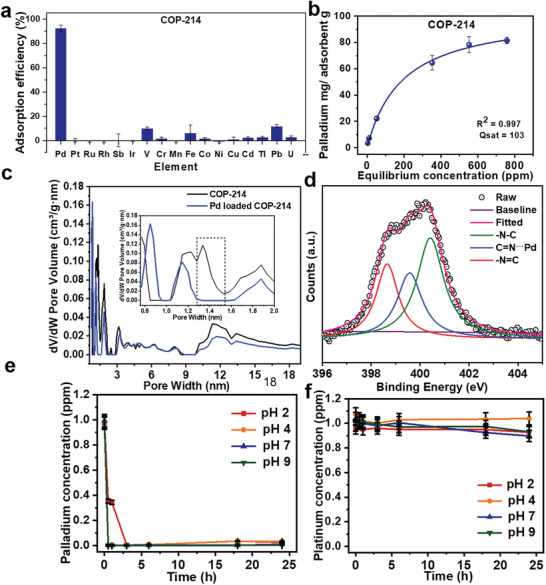
a) Precious metal adsorption selectivity of COP‐214 in a mixture of metals containing precious metals (Pd, Pt, Ru, Rh, Sb, Ir) and transition metals (V, Cr, Mn, Fe, Co, Ni, Cu, Cd, Tl, Pb, U). b) Palladium adsorption isotherms of COP‐214. The adsorption isotherms were measured by dissolving metal salts (K_2_PdCl_4_) in deionized water (DIW) without buffering agents. c) Comparison of pore‐size distribution of COP‐214 and Pd‐loaded COP‐214 according to the NLDFT. The inset graph shows the dramatic change of the micropores from ≈1.3 to ≈1.5 nm due to pore filling during Pd capture. d) XPS spectra of N1s of COP‐214 after Pd loading. Data analysis and quantification were performed using Origin 9.60 software. A Lorentz Model was used in the curve fitting of the spectra. The full width at half maximum of all peaks was kept between 1.1 and 1.3 eV for consistency. e) pH effect of COP‐214 on Pd adsorption. f) pH effect of COP‐214 on Pt adsorption. (0.1 m HCl and 0.1 m NaOH solutions were used to adjust the pH of the solution to 2, 4, 7, and 9. The study of the pH effect was analyzed by ICP‐MS at different time intervals). All experiments were performed at least three times and data are denoted as average values. Bars correspond to standard errors of triplicate samples.

Interestingly, COP‐214 exhibited good potential for the selective capture of Pd even in the presence of other strongly coordinating metals, with negligible uptake (Table S6, Supporting Information) of V, Fe, Pd, U, Tl, and Cd (**Figure** **4**a). To the best of our knowledge, COP‐214 is the first heterogeneous adsorbent that shows selective Pd separation in the presence of Pt.

**Figure 5:**
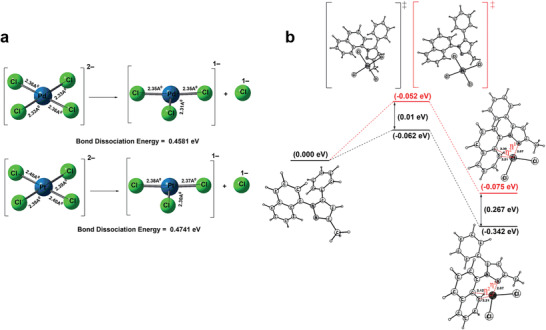
DFT calculations for Pd and Pt adsorption on COP‐214: a) Bond dissociation energy was calculated to understand the ligand exchange process. The bond dissociation energy for [PtCl_4_]^2−^ conversion into [PtCl_3_]^−^ + Cl^−^ is 0.016 eV (10.04 kcal mole^−1^) higher than for the corresponding [PdCl_4_]^2−^ conversion. b) Complexation with N‐sites of COP‐214. A single COP‐214 unit was used to determine the binding affinity of Pd and Pt in COP‐214, where the metals coordinate at the pyrazole units and the “dotted red line” denotes dative bonds. The results confirm that Pd forms a more stable complex at a lower binding energy (−0.342 eV) through a transition state energy of −0.062 eV, whereas the Pt complex is less energetically favorable (−0.075 eV) and, with a higher transition state energy (−0.052 eV), more prone to a backward process.

#### 2.2.1 Experimental Study for Pd Binding on COP‐214

To investigate the selective uptake of Pd in more detail, we carried out experiments on equilibrium uptake capacity and on pH effects. The maximum uptake of Pd in COP‐214 was found to be 103 mg g^−1^ (Figure 4b) from the Pd adsorption isotherms using standardized solutions in batch conditions and fitted by the Langmuir adsorption isotherm model. After Pd uptake, Pd‐loaded COP‐214 showed a decrease in surface area (132 m^2^ g^−1^) when compared to the parent COP‐214 (308 m^2^ g^−1^). The micropores in the 1.4–1.5 nm region, in particular, were blocked after metal capture, possibly due to the pore filling behavior of COP‐214 (Figure S15, Supporting Information). These observations confirm that Pd ions are well‐suited to fit in the micropores of COP‐214 (Figure 4c).

Yun and co‐workers showed that an MOF with 6‐connected Zr_6_‐nodes was suitable for palladium (PdCl_4_^2−^) recovery. This could be attributed to the optimum size of PdCl_4_^2−^ ions for fitting into and diffusing through the pores of MOF structures. Pd forms a stable divalent chloride complex anion of PdCl_4_^2−^ (5.5 Å) in acidic conditions and in the presence of chloride anions, and its size enables it to fit into or diffuse through the pore of COP‐214 (15 Å).^[^ ^50^ ^]^

The powder X‐ray diffraction data of Pd‐loaded COP‐214 exhibited a broad pattern due to the amorphous nature of the porous polymer with no elemental Pd peaks, confirming the absence of any reductive mechanism (Figure S16, Supporting Information) present in other precious metal studies.^[^ ^51^, ^52^, ^53^ ^]^ Elemental analysis of Pd‐loaded COP‐214 also shows Pd (5.67 wt%) trapped inside the structure (Table S7, Supporting Information).

TEM analysis and elemental mapping also confirm the selective sorption of Pd (Figure S20, Supporting Information). The structural‐element (C, N) distribution in the Pd‐loaded COP‐214 reveals the integrity of its structures, and the uptake of metal is confirmed by the appearance of Pd uniformly throughout the surface. The bright spots observed on the high‐angle annular dark‐field image are mainly due to the adsorbed electron‐rich Pd metal on the COP‐214 surface through an even dispersion.

To gain more insight into the mechanism, XPS analysis was carried out after Pd uptake. In XPS, Pd‐loaded COP‐214 showed doublet peaks (337.22 and 342.52 eV) in the Pd(II) range^[^ ^54^ ^]^ for Pd 3d_5/2_ and 3d_3/2_, which could be attributed to the presence of Pd(II) bound within the porous polymer networks (Figure S18a, Supporting Information). After Pd loading, the N1s signal for the fitted data shows (Figure 4d) three peak patterns instead of two (Figure 3b). The following N1s peak locations were observed: —N—C (400.41 eV), −N=C (398.65 eV), and N‐coordinated to Pd—N (399.57 eV). After binding with Pd, a new peak emerged at 399.57 eV, due to the physiochemical interaction of Pd (II) with N‐sites (398.65 eV) (Figure 4d).^[^ ^55^ ^]^ The areas of these three signals also indicate varying quantities of different N1s environments in COP‐214. After Pd capture, the imine (—C=N) of N1s was split due to interaction with Pd. These phenomena prove that selective Pd capture happens due to micropore trapping of the metal ions and physiochemical interactions of pyrazole moieties with N‐sites.

The adsorption behaviors of Pd and Pt were also monitored in parallel and under a wide range of pH conditions. Surprisingly, palladium shows very rapid and effective adsorption at varying pH (Figure 4e), whereas COP‐214 does not capture Pt at any pH (Figure 4f).^[^ ^56^ ^]^ The pH was controlled using HCl and NaOH solutions. COP‐214 does not uptake Pt even after 25 h of testing under various pH conditions. This remarkable selectivity of COP‐214 may be a promising application for Pd capture from precious metal solutions. Reusability is an essential characteristic of a good adsorbent, which should possess stable adsorption and desorption performance throughout its life‐cycle (in the event that an appropriate desorption agent is required). To optimize the desorption conditions for the adsorbed Pd on the COP‐214, four different desorption solutions were tested (Table S8, Supporting Information). First, the loaded Pd(II) was eluted from COP‐214 with acidic thiourea (0.1 m SC(NH_2_)_2_ and 1 m HCl) desorption solutions. After shaking for 3 and 24 h, the mixtures were filtered using a syringe filter and the metal concentrations were analyzed by ICP‐MS. The Pd desorption efficiencies were calculated using the initial adsorption amounts per gram of adsorbent. COP‐214 could be used up to four cycles and over 94% desorption efficiency was observed during a recyclability study (Figure S19, Supporting Information). It is notable that the amount of Pd increased in subsequent steps due to the additional recovery of previously captured Pd in COP‐214. Table S9, Supporting Information, shows a comparison of the selective Pd(II) recovery of different adsorbents. Zr‐based MOF^[^ ^50^ ^]^ exhibits selective‐adsorption behavior in mixed‐metal solutions of Co (II), Ni(II), Cu(II), Zn(II), and Pd(II), but not Pt (II). Considering selectivity, capacity, and ease of preparation, COP‐214 is the best adsorbent available for the selective capture of Pd(II) in the presence of competing ions, most notably Pt(II).

#### 2.2.2 Theoretical Study for Pd Binding on COP‐214

DFT calculations were carried out to investigate Pd (II)‐binding behavior in the pores of COP‐214. All the DFT geometry optimizations were performed by a Gaussian 16 program package.^[^ ^57^ ^]^ The electronic exchange and correlated energy contributions to the total electronic energy were approximated using a B3LYP2^[^ ^58^ ^]^ hybrid functional. All intermediate and transition state geometries were optimized with a 6–311G** basis set.^[^ ^59^ ^]^ A single COP‐214 representative unit was used to calculate the binding energy (*E*_bind_) of Pd and Pt ions in COP‐214, where the metals approach the N‐sites of the pyrazole units.

The first step in the calculation was to prepare a coordinatively unsaturated precious metal. The bond dissociation energy was calculated for removing a Cl^−^ ion from the PdCl_4_^2−^ and PtCl_4_^2−^ to understand the feasibility of the ligand exchange process. The result shows that the bond dissociation energy for [PtCl_4_]^2−^ (into [PtCl_3_]^−^ + Cl^−^) is 0.016 eV (10.04 kcal mole^−1^) higher than for [PdCl_4_]^2−^.^[^ ^60^ ^]^Next, the dechlorinated species were studied for interaction with the *η* ^2^ aromatic ring and *η* ^1^ N‐sites of the pyrazole units.^[^ ^61^, ^62^ ^]^ Calculations show that Pd forms a more stable complex at a lower binding energy (−0.342 eV) through a favorable transition state energy of −0.062 eV, whereas the Pt complex is energetically more demanding (−0.075 eV) and requires a higher transition state energy (−0.052 eV) with a clear tendency for a reverse process (**Figure** **5**). These theoretical calculations are consistent with the experimental observations, where Pd selectivity is observed through both steric and electronic factors.

## 3 Conclusion

In summary, we demonstrated a novel, efficient, and scalable synthesis of pyrazole‐based porous polymers with large surface areas without any metal catalysis. Notably, we proved that polymers are the kinetically favored product of *syn* addition geometry using comparisons with the single‐crystal structure of a model compound. The polymers show high thermal stability up to 463 and 425 °C under nitrogen atmosphere and air, respectively. These materials also show exceptional stability in various solvents such as H_2_O, THF, and CH_2_Cl_2_, and in harsh environments such as HCl or NaOH for over two weeks. They also showed very high CO_2_ separation performance under flue gas and landfill gas conditions. Remarkably, COP‐214 showed a rapid and selective removal of Pd from a precious metal mixture in an acidic medium. It can be reused for up to four cycles with >94% efficiency. The Pd adsorption mechanism of COP‐214 was investigated using XRD, XPS, TEM, and BET surface area analyses, and simple physisorption was found to be prevalent. Furthermore, the binding energies (*E*_bind_) of PdCl_4_^2−^ with COP‐214 calculated through the DFT resulted in a much lower *E*_bind_ for PdCl_4_^2−^ indicating a strong complexation with COP‐214.

## Experimental Section

##### Synthesis of COP‐214

Pyrazole‐based COP‐214 was prepared using a double Schiff reaction between 0.053 mmol (**2**) (30 mg) and 0.08 mmol amines (**4**) (15 mg) in a sealed Pyrex tube in a 1,4 dioxane:mesitylene (4:3 mL) system through the ampoule method. The reaction mixtures were transferred into a Pyrex tube and sonicated for 15 min. The mixtures were degassed under liquid N_2_ (77K) by freeze‐pump‐thaw cycles four times. The Pyrex tube was sealed and the reaction mixture was allowed to reach room temperature. The sealed Pyrex tube containing the reaction mixture was kept at 120 °C with a heating rate of 2 °C per min for 3 d. The precipitate was washed thoroughly with dioxane, water, and acetone, and dried at 100 °C under vacuum overnight to obtain a 56% isolated yield. COP‐214‐MW was synthesized using microwaves using the same starting monomers and solvent mixtures. The resulting mixture was exposed to microwave irradiation at 120 °C for 2 h. After collecting the solid brown‐colored precipitate, purifications were carried out following the same methods used for the conventional heating product.

##### Synthesis of Model Compound 6

The model compound (**6**) was prepared by first dissolving 1‐phenylbutane‐1,3‐dione (**5**) (0.17 gm, 1.06 mmol) and 1,5 naphthalene di‐hydrazine (**4**) (0.1 gm, 0.53 mmol) in ethanol (30 ml). The resulting mixture was refluxed for 24 h and cooled down. The solvent was evaporated and a brown‐colored solid (yield 68%) was obtained. Finally, the model monomer was crystallized from a DMF and acetonitrile solvent mixture. The model compound crystals were carefully characterized by ^1^H, ^13^C NMR, 2D ^1^H NOESY, and 1D ^1^H NOE spectra; and LC‐MS and single‐crystal diffraction. Anal. Calcd. for (C_30_H_24_N_4_): C, 81.79; H, 5.49; N, 12.72. Found: C, 81.42; H, 5.25; N, 11.99.

##### Sorption Experiments—Metal Selectivity Analysis

The multi‐element standard solutions (10 ppm for each metal) for ICP‐MS were diluted to 100 ppb with the addition of DIW. 10 mg of adsorbent (COP‐214) was added to 10 mL of the solution. Three experimental samples and two control samples were prepared and treated in the same manner. The mixtures were shaken at 8 rpm for 24 h and filtered using a 0.50 µm syringe filter. The metal concentrations were measured by ICP‐MS. The adsorption amounts were measured by a comparison of the control and experiment concentrations.

##### Sorption Experiments—Pd Adsorption Isotherm

10, 50, 100, 500, 750, and 1000 ppm of Pd solutions were prepared from stock solutions of K_2_PdCl_4_. 10 mg of adsorbent was added to 10 mL of the solution at each ppm. The mixture was shaken at 8 rpm for 48 h and filtered using a 0.50 µm syringe filter. After 48 h, the polymer was collected by filtration with filter paper and washed thoroughly with DIW. The metal‐loaded polymers were dried in a vacuum oven at 100 °C for overnight. The metal concentrations in filtrates were measured by ICP‐MS and compared with the initial concentrations to calculate the adsorbed metal amounts.

##### Sorption Experiments—Study of the pH Effects on Pd and Pt Adsorption

To investigate the metal adsorption behavior of COP‐214 at varying pH, the following experiments were performed. The Pd and Pt stock solutions were prepared by dissolving K_2_PdCl_4_ and K_2_PtCl_4_ in DIW. The stock solution was diluted to 1 ppm with the addition of DIW, 0.1 m HCl, and 0.1 m NaOH solution to adjust the pH of the solution to 2, 4, 7, and 9, respectively. 10 mg of COP was added to 100 mL of the prepared solutions at each pH. The mixture was magnetically stirred and after 0.5, 1, 3, 6, 18, and 24 h, ≈1 to ≈3 mL of solution was taken and filtered using a syringe filter. The remaining metal concentrations in the solutions were measured by ICP‐MS.

##### Sorption Experiments—Desorption Conditions and recyclability

A Pd solution at 1000 ppm was used for the adsorption process and the adsorption was carried out as discussed earlier. For metal desorption, 100 mL of 0.1 m SC(NH_2_)_2_ and 1 m HCl desorption solutions were used. 10 mg of Pd‐adsorbed COP‐214 was added to 10 mL of desorption solution. The mixture was placed in the heating mantle at 40 °C and stirred for 3 h. The adsorbent was separated by filtration and washed with DIW. The Pd concentration in the filtrate was measured by ICP‐MS, and the desorption efficiency was calculated based on the Pd adsorption amount. After drying fully in a vacuum, the adsorbent was reused for the adsorption, and the ad–desorption processes were repeated.

##### Crystallographic Data (CIF)

CCDC 1996618 contains the supplementary crystallographic data for this paper. These data can be obtained free of charge from The Cambridge Crystallographic Data Centre via www.ccdc.cam.ac.uk/data_request/cif.

## Conflict of Interest

The authors declare no conflict of interest.

## Author Contributions

M.G. and M.M. contributed equally to this work. C.T.Y. conceived and supervised the project. M.G. and M.M. carried out all synthesis and testing of pyrazole porous polymers for selective Pd recovery. Y.H. carried out ICP‐MS analysis. V.R. contributed to the monomer design and synthesis. U.J. assisted with the analysis. Z.U. provided DFT calculations. All authors discussed the results and commented on the manuscript.

## Supporting information

Supporting Information

## Data Availability

Research data are not shared.
