# Supplementary material for: Asynchronous Double Schiff Base Formation of Pyrazole Porous Polymers for Selective Pd Recovery
Source: Adv Sci (Weinh). 2021 Mar 2;8(8):2001676. doi: 10.1002/advs.202001676 (PMC8061357; doi:10.1002/advs.202001676)
Supplement: Supplementary file 1 — Supporting Information [file ADVS-8-2001676-s001.pdf]

WILEY-VCH

© 2021 Wiley-VCH GmbH

ADVANCED SCIENCE
Open Access

Supporting Information

for *Adv. Sci.,* DOI: 10.1002/advs.202001676

Asynchronous Double Schiff Base Formation of Pyrazole Porous Polymers for Selective Palladium Recovery

*Mousumi Garai, Manmatha Mahato, Yeongran Hong, Vepa Rozyyev, Uiseok Jeong, Zakir Ullah and Cafer T. Yavuz**

Supporting Information

Asynchronous double Schiff base formation of pyrazole porous polymers for selective Palladium recovery

*Mousumi Garai[§], Manmatha Mahato[§], Yeongran Hong, Vepa Rozyyev, Uiseok Jeong, Zakir Ullah and Cafer T. Yavuz**

Dr. M. Garai, Y. Hong, U. Jeong, Prof. C. T. Yavuz
Department of Chemical and Biomolecular Engineering, Korea Advanced Institute of Science and Technology (KAIST), 291 Daehak-ro, Yuseong-gu, Daejeon 34141, Korea
(*) Email: yavuz@kaist.ac.kr, cafer.yavuz@kaust.edu.sa

Dr. M. Mahato, V. Rozyyev, Prof. C. T. Yavuz
Graduate School of Energy, Environment, Water and Sustainability (EEWS), KAIST, Daejeon 34141, Korea

Zakir Ullah, Prof. C. T. Yavuz
Department of Chemistry, KAIST, Daejeon 34141, Korea

Prof. C. T. Yavuz
Advanced Membranes and Porous Materials Center (AMPM), Physical Sciences and Engineering (PSE), King Abdullah University of Science and Technology (KAUST), Thuwal 23955-6900, Saudi Arabia

(§) Equal first authorship

Contents:

1. Experimental details

2. Structural and morphology characterization

3. X-ray crystallography of 6

4. Elemental composition of palladium adsorbed COP-214 and desorption efficiencies of COP-214

5. Theoretical methodology

6. References

1. Experimental details

1.1. Materials

The chemicals acetyl chloride and boron trifluoride-acetic acid-complex and 1,5-diaminonaphthalene were purchased from Sigma Aldrich. All solvents were purchased from Samchun and used without further purification. Metal selectivity testing was done by using multi-element calibration standard solutions of ICP-MS for each metal (Pd, Pt, Ru, Rh, Sb, Ir, V, Cr, Mn, Fe, Co, Ni, Cu, Cd, Tl, Pb, U) from Agilent (Part No. 8500-6948 and 8500-6940). For other analyses like pH effect, adsorption isotherms, metal desorption and reusability testing were performed by potassium tetrachloropalladate (II) (K_2PdCl_4, 98 %), and potassium tetrachloroplatinate (II) (K_2PtCl_4, 98 %). Potassium tetrachloropalladate (II) (K_2PdCl_4, 98 %), and potassium tetrachloroplatinate (II) (K_2PtCl_4, 98 %) were purchased from Merck. For all metal adsorption and desorption experiment, deionized water (DIW) obtained from Mili-Q (18.2 MQ·cm at 25°C) system was used.

1.2. Methods

Synthesis of monomer units

The monomer units acetylacetonate tris-phenyl benzene (**2**) and 1,5-naphthalene di-hydrazine (**4**) were prepared following reported methods.[S1-S3]

1.3. Analytical method

The products were confirmed from ^{1}H, ^{13}C NMR, 2D ^{1}H NOESY and 1D NOE spectra using $CDCl_3$/D^{6} DMSO as the solvents and Bruker AVANCE 300, 400 MHz as the equipment. Fourier transform infrared (FT-IR) spectra were recorded on a SHIMADZU IRTracer-100 spectrometer with GladiATR 10 (single reflection ATR accessory) in the 400–4000 cm^{-1}. Thermogravimetric analyses were performed on a Shimadzu DTG-60A by heating the samples up to 800°C at a rate of 10°C min^{-1} under nitrogen or air atmosphere. X-ray

photoelectron spectroscopy (XPS) was carried out on a K-alpha model of Thermo VG Scientific equipped with a micro focused monochromator X-Ray source with the energy resolution of 0.5 eV full-width at half-maximum under ultrahigh vacuum condition of 10^{-9} Torr. In order to evaluate the porosity of the polymers, adsorption isotherms were obtained on a Micromeritics 3FLEX accelerated surface area and porosimetry analyzer at 77~87K. Prior to the measurements using argon or nitrogen, samples were degassed at 110°C for 12 hrs under vacuum. The specific surface areas were calculated by Brunauer-Emmett-Teller (BET) method. Pore size distribution was measured using NLDFT model assuming slit pore shape. CO_2 and N_2 adsorption–desorption isotherms were measured at 273, 298 and 323K. A temperature controller was used to maintain the temperature during adsorption/desorption analysis. The CO_2/N_2 selectivities were calculated through ideal adsorbed solution theory (IAST) from the CO_2 and N_2 adsorption isotherms at three temperature points (273, 298 and 323 K) by using Mathematica software. The CH_4 uptake was measured at 273 K by using a static volumetric system (3Flex surface characterization analyzer, Micromeritics Inc.). The adsorption and desorption temperature were kept constant by using a proportional–integral–derivative (PID) controller. In case of H_2 uptake, the analysis was done at 77K in a liquid N_2 bath. The standard volumetric technique was used to obtain the sorption data of gas molecules within the pressure range up to 1200 mbar. The quantity of adsorption was recorded from the instrument in mmol/g. IAST calculations were carried out by fitting adsorption isotherms with either a single-site Langmuir model or a dual-site Langmuir model. Selectivity values predicted by IAST for CO_2/CH_4=50/50 and CO_2/N_2 =15/85 mixture at 273 K with increasing pressure (bar) for COP-214 and COP-214MW. Isosteric heat of absorption (Qst) values were determined by using the Clausius−Clapeyron equation, again on Mathematica. Qst values were determined using three different temperatures to be more accurate."

The metal concentrations were measured by ICP-MS instrument (Agilent, 7700x). Elemental analysis for C, H, N and O was conducted using a FLASH 2000 series of Thermo

Scientific. Powder X-ray diffraction (PXRD) measurement was carried out over the 2θ range of 5-90° using a SmartLab from Rigaku. Crystal structure analysis was determined by single-crystal diffraction methods at the Korea Basic Science Institute (KBSI, Western Seoul Center, Seoul, Korea). The single crystal data was collected on a Bruker SMART CCD diffractometer equipped with a graphite-monochromated Mo Kα (λ = 0.71073 Å) radiation source and a nitrogen cold stream (200(2) K). Data collection and integration was performed on a SMART (Bruker, Madison, WI, USA, 2000) and SAINT-Plus (Bruker, 2001)[S4]. Absorption correction was performed by a multi-scan method implemented in SADABS[S5]. The structure was solved by direct methods and refined by full-matrix least-squares on F^2 using SHELXTL[S6]. All the nonhydrogen atoms were refined anisotropically, and hydrogen atoms were fixed to their geometrically ideal positions.

2. Structural and morphological characterization

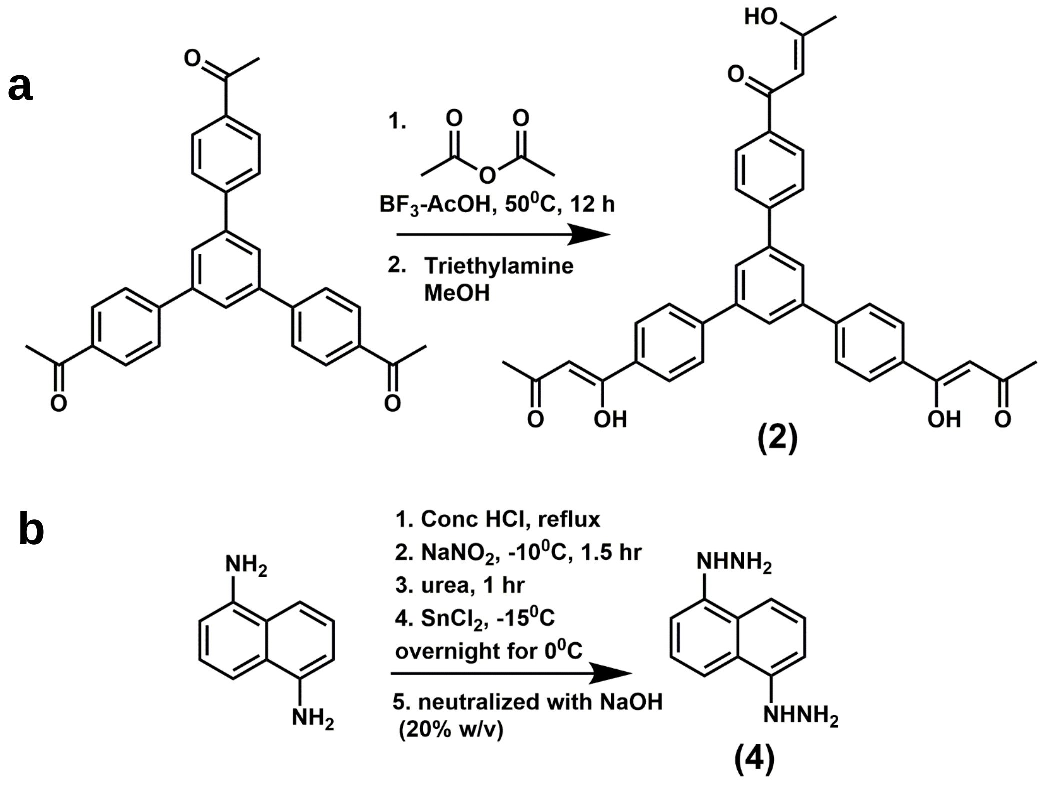

Figure S1. Syntheses of (a) **2** and (b) **4** from commercially available monomers. (The yield of **2** and **4** is 78% and 54% respectively).

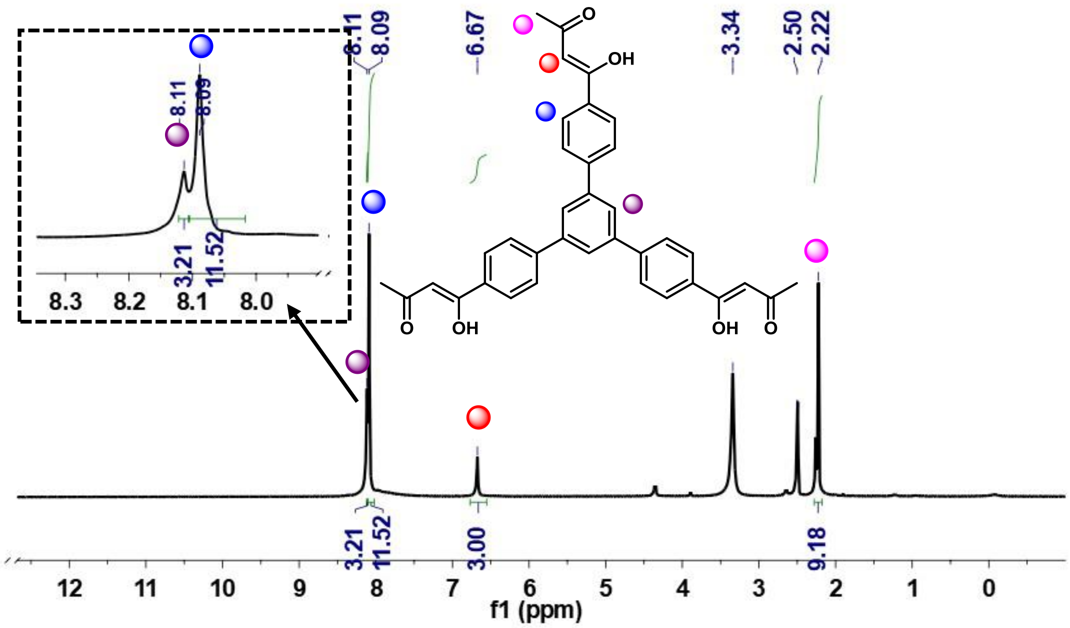

Figure S2. ^{1}H NMR (D[6] DMSO, 298 K) of **2**. The two types of aromatic protons are clearly denoted at 8.11 ppm (central phenyl protons, 3H) and 8.09 ppm (outside phenyl groups, 12H) (inset graph). -C=CH protons of the acac units (3H) appear at 6.67 ppm. The side anchoring –CH_3 groups (9 H) are present at 2.22 ppm.

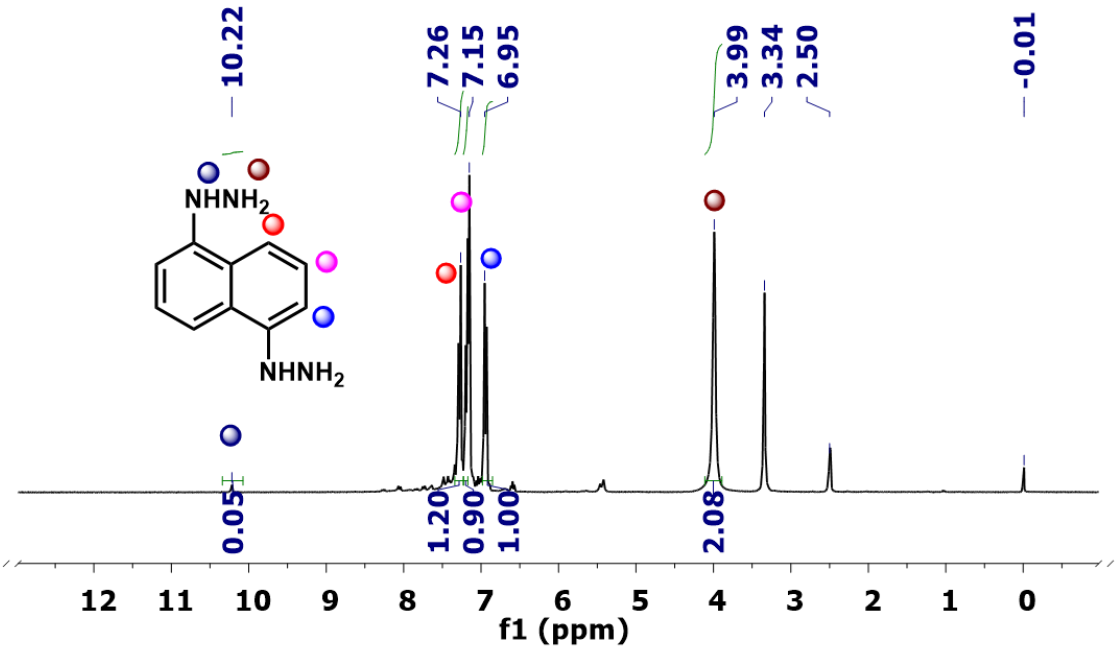

Figure S3. ^{1}H NMR (D[6] DMSO, 298 K) of **4**. δ_{ppm} 10.22 (-NH, small peak), 7.26 (Aromatic-H, d, 2H), 7.15 (Aromatic-H, m, 2H), 6.95 (Aromatic-H, d, 2H), 3.99 (-NH_2, s, 4H).

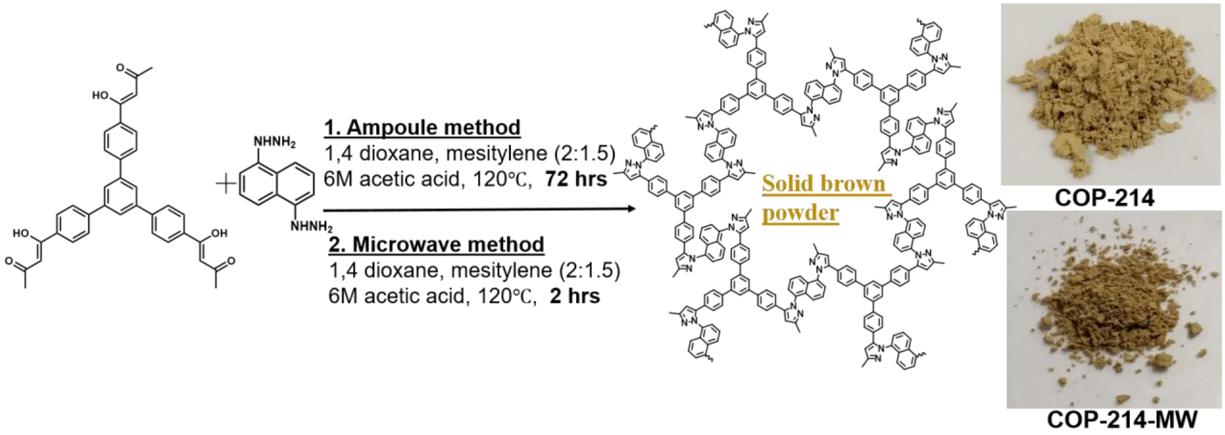

Figure S4. Synthetic scheme of COP-214 via ampoule and microwave syntheses. Reaction condition: 1,4-dioxane: mesitylene (2:1.5), 6M acetic acid, 120 ℃, 72 hrs and 2 hrs respectively. The image of brown color product is shown in right side.

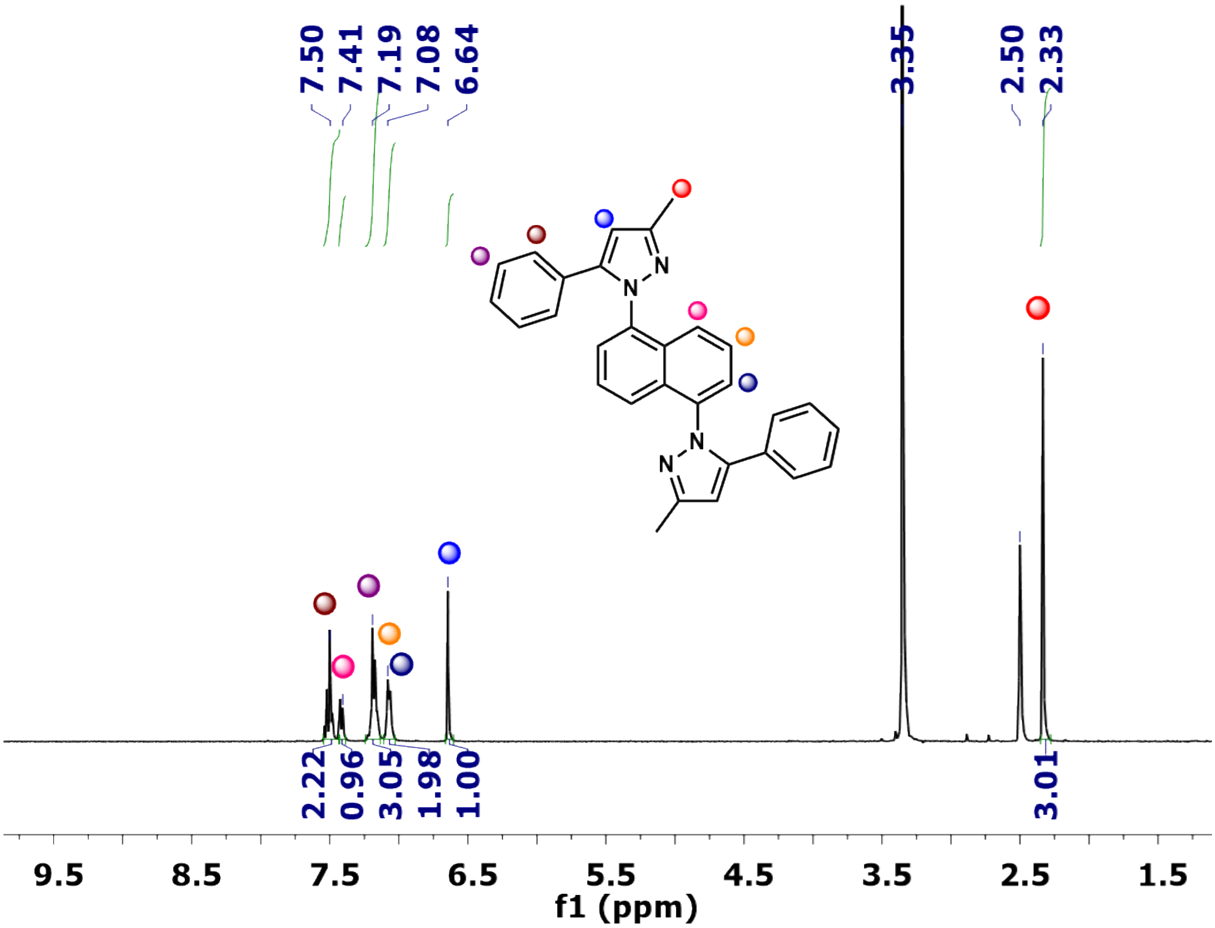

Figure S5. ^{1}H NMR (D^{6} DMSO, 298 K) of **6**. δ_{ppm} 7.50 (d, J=7.51 Hz, 4H, benzene), 7.41 (d, J=7.43 Hz, 2H, benzene), 7.19 (m, 6H, benzene), 7.08 (d, 7.08 Hz, 4H, benzene), 6.64 (s, 2H, CH), 2.33 (s, 6H, CH_3),

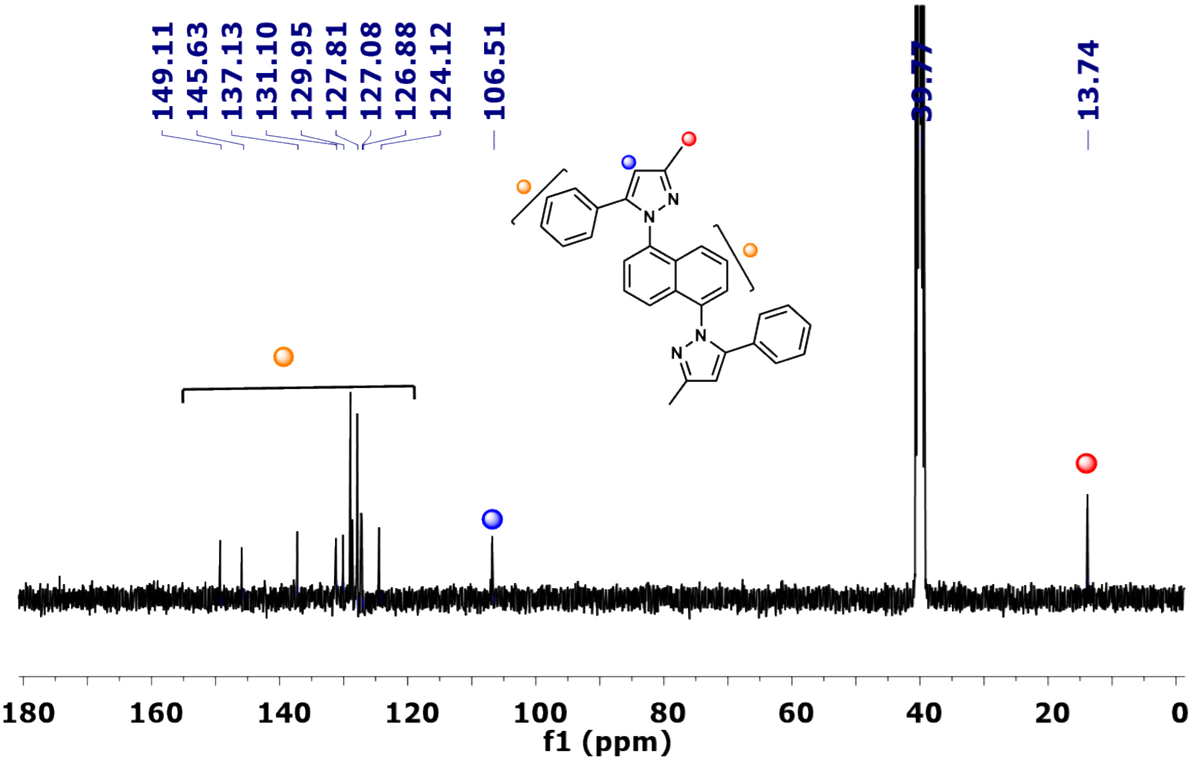

Figure S6. ^{13}C NMR (D[6] DMSO, 298 K) of **6**. δ_{ppm} 149.11, 145.63, 137.13, 131.10, 129.95, 127.81, 127.08, 126.88, 124.12, 106.51, 13.74.

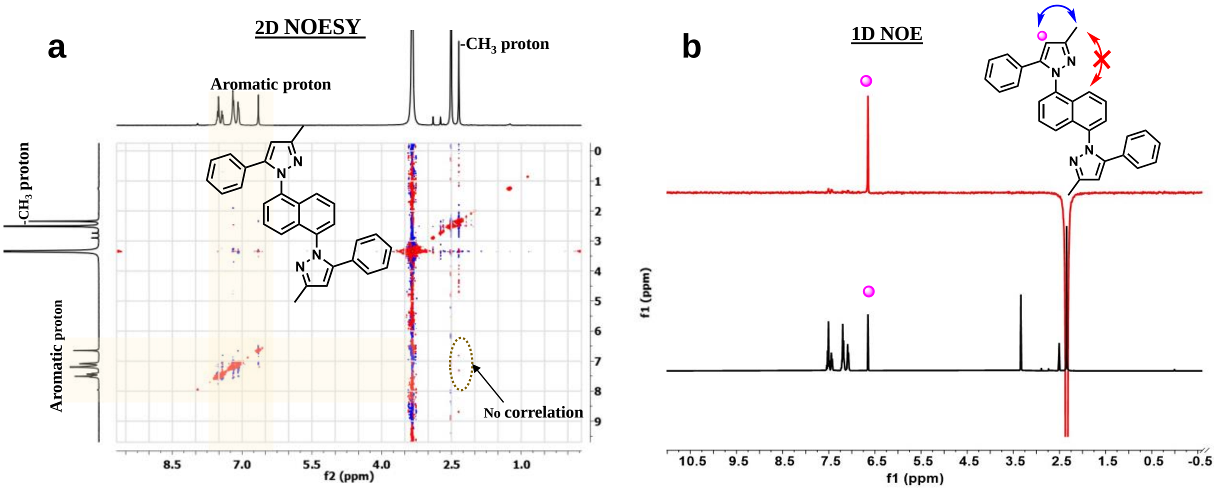

Figure S7. (a) 2D NOESY spectra of **6** found no correlation of –CH_3 group with aromatic protons indicating that –CH_3 group is not in close proximity to the aromatic moieties. Some correlation of aromatic region could be attributed to benzene units being close to each other. (b) 1D NOE spectra of **6** under irradiation of specific –CH_3 protons showing reverse direction (top figure, red color), only –CH proton of acac units appear due to close proximity of –CH_3 group and aromatic region peaks are not visible. This confirms the NOESY experiments that methyl group is not in close proximity of the aromatic units.

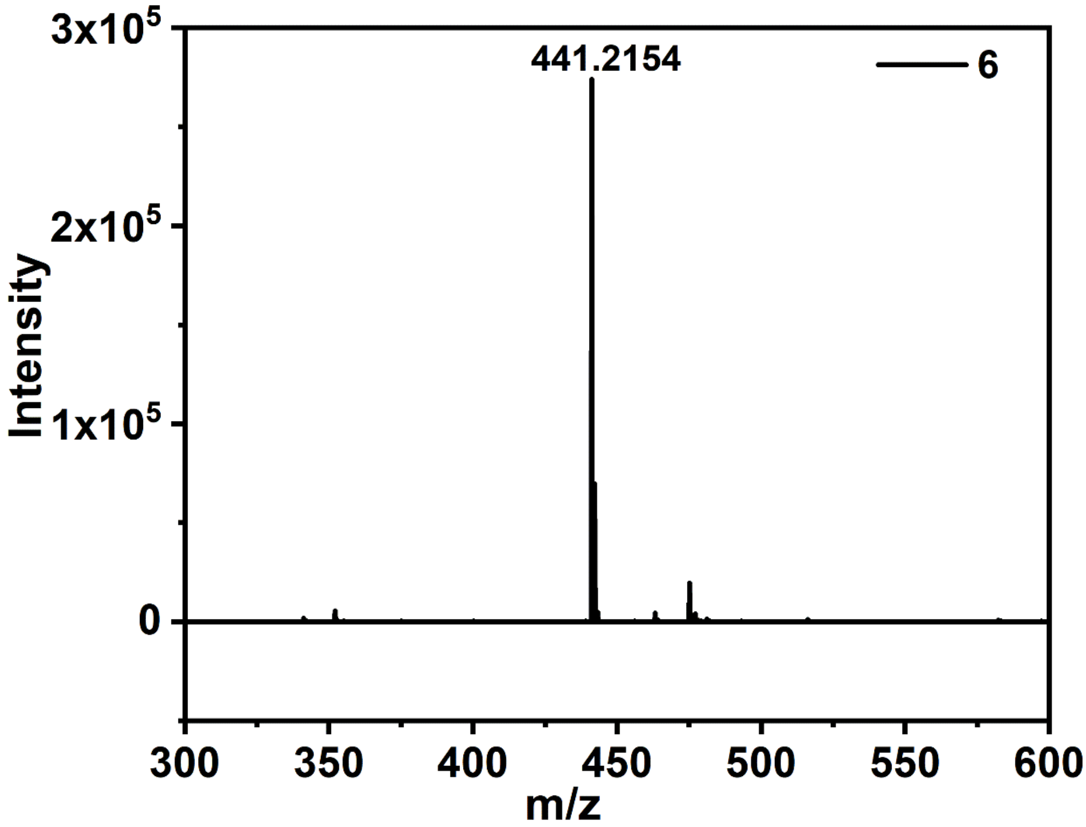

Figure S8. LC-MS of model compound **6** in acetonitrile solvent confirming pyrazole formation (m/z: 441.2154).

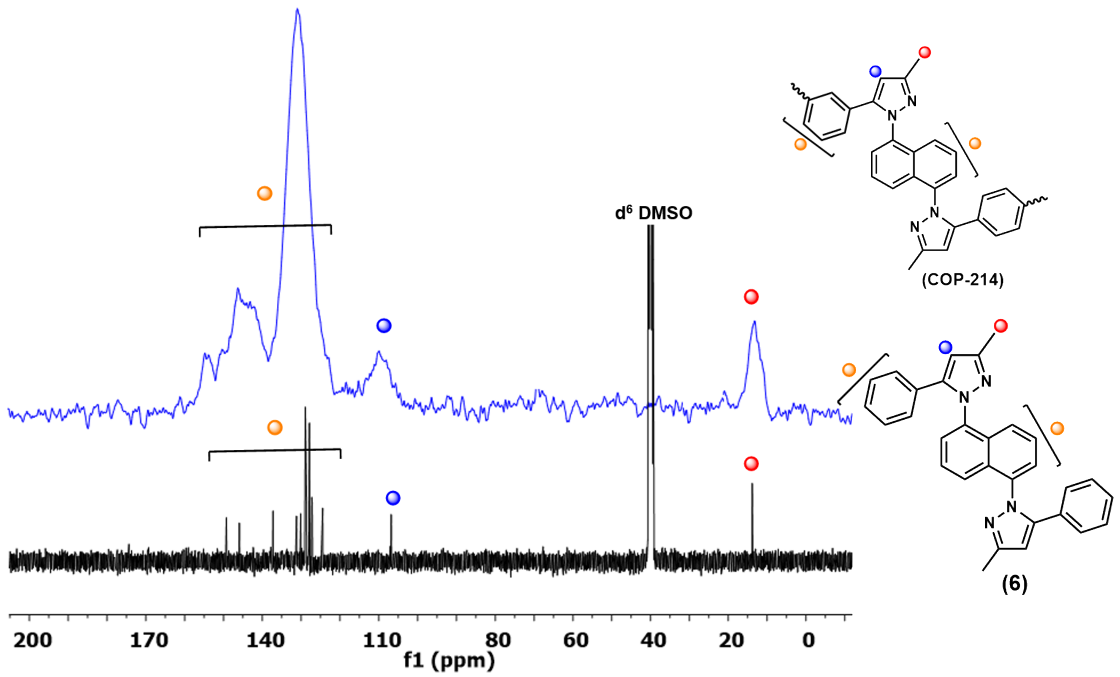

Figure S9. ^{13}C CP/MAS solid-state NMR spectra of COP-214 (top, blue) and ^{13}C NMR spectra of model compound **6** (bottom, black). The carbon atom of the –C=CH and $–CH_3$ of acetylacetonate unit resonated at 13.7 and 106.5 ppm respectively, confirming the presence of acetylacetonate fragment and aromatic moieties carbon peaks also show good correlation with model compound.

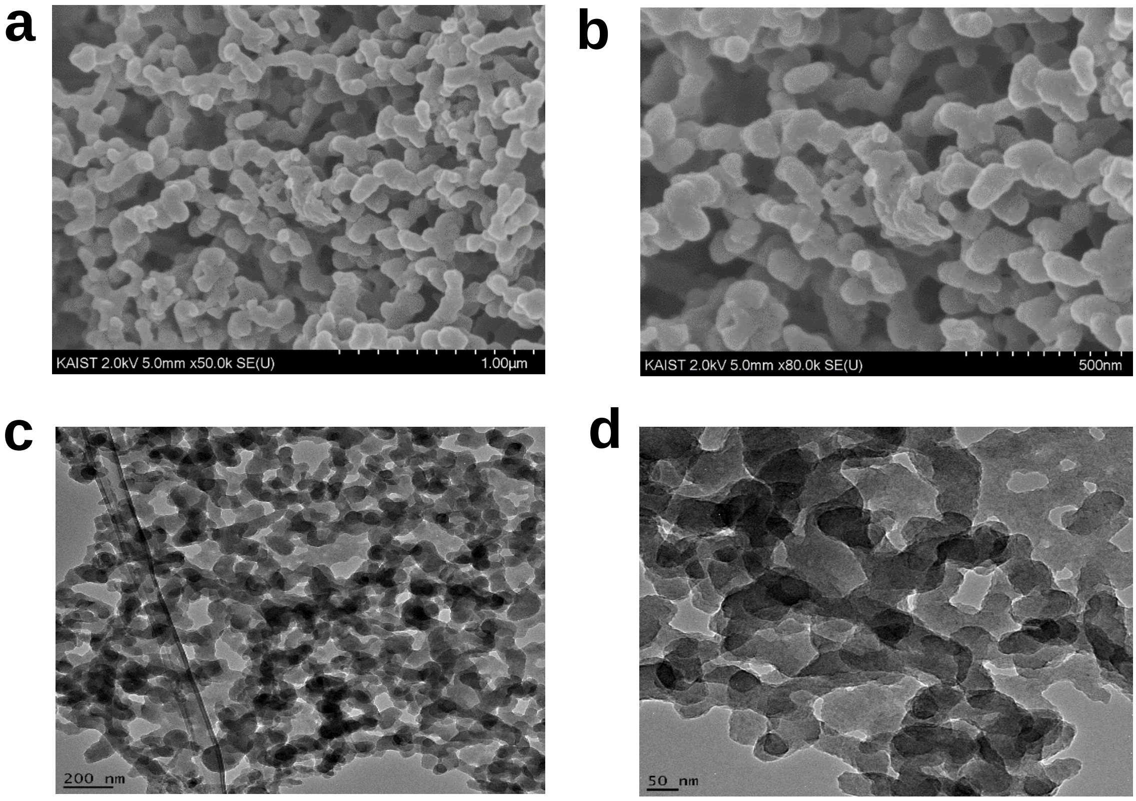

Figure S10. Morphology analysis of COP-214: SEM images magnification scale bar (a) 1 μm, (b) 500 nm; TEM images magnification scale bar (c) 200 nm, (d) 50 nm.

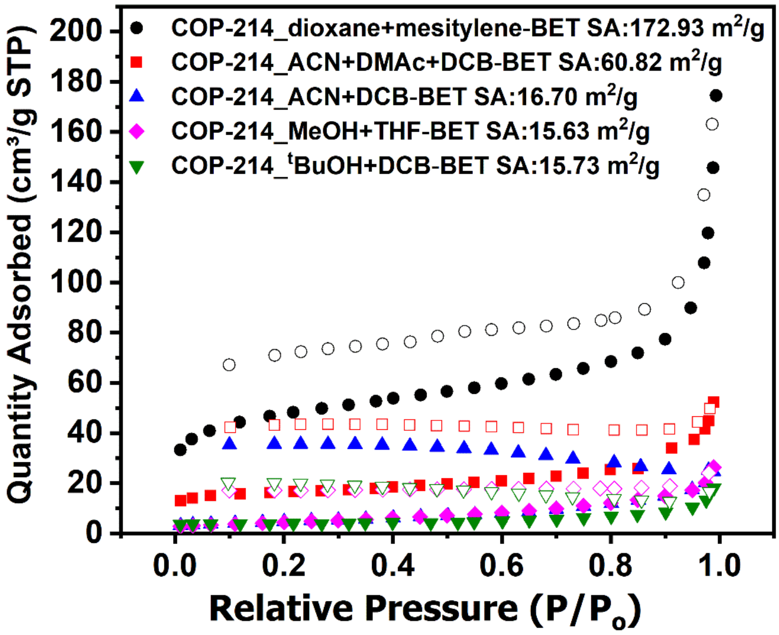

Figure S11. Nitrogen adsorption-desorption isotherms and BET surface areas of COP-214 under solvent screening. A combination of dioxane and mesitylene of COP-214 shows good porosity (172 m^2/g). This solvent system is chosen for further gas capture and metal recovery experiments.

2.1. Gas Sorption, Selectivity Studies of COP-214

The CO_2 uptake is rapid even at lower pressures, proving that pyrazole moieties have favorable interactions. CO_2 uptake capacity of COP-214 is 1.3 mmol g^{-1} at 273 K, 0.73 mmol g^{-1} at 298 K, and 0.38 mmol g^{-1} at 323 K, respectively (Figure S12a). It has been observed that the electron-rich nitrogen-containing porous polymers show strong dipole–quadrupole interaction with CO_2, leading to a significant increase in the CO_2 uptake.[S7,S8] At 273 K, COP-214 shows a predictable uptake of CH_4 (0.36 mmol.g^{-1}), and N_2 (0.05 mmol.g^{-1}) (Figure S12c). At 77 K, COP-214 posts a H_2 uptake of 2.50 mmol.g^{-1}, whereas COP-214-MW shows 2.54 mmol.g^{-1} (Figure S12d).

High selectivity of CO_2 over N_2 is one of the key appearances for an adsorbent to be effective for CO_2 capture. CO_2/N_2 selectivity for landfill/flue gas conditions (CO_2/N_2 = 15:85) was determined according to the standard ideal adsorbed solution theory (IAST). The CO_2/N_2 selectivity of COP-214 at 1 bar is 102 at 273 K (Figure S13a), which was higher than many porous polymers,[S9] azo-linkage polymers,[S10] conjugated mesoporous polymers[S11] and ultramicroporous benzothiazole polymers[S12] and also suggest that COP-214 can be useful in this context.

To study the performance for CH_4 purification, we examined the selectivity of COP-214 for CO_2/CH_4 with the IAST method under gas feed compositions of 5:95 for natural gas sweetening and 50:50 for landfill gas purifications. COP-214 shows selectivities for gas composition ratios 9.6 (5:95) (Figure S13a) and 9.8 (50:50) at 273 K (Figure S13b).

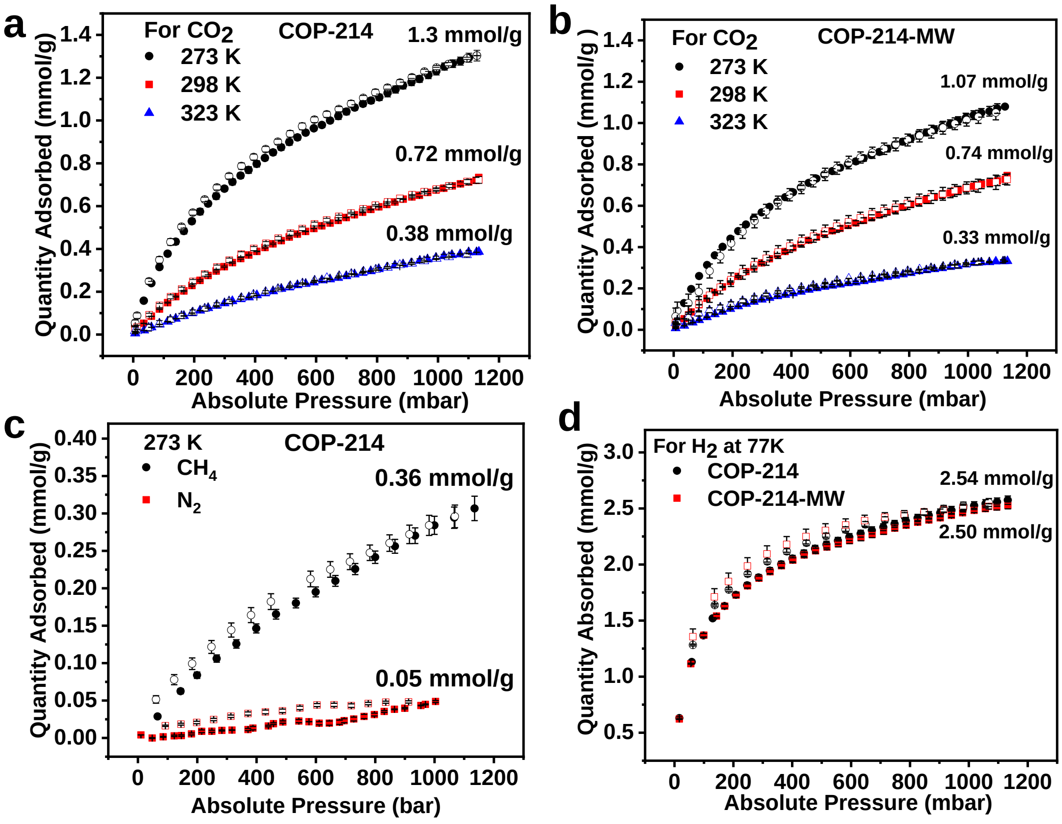

Figure S12. Gas adsorption properties: (a) CO_2 adsorption of COP-214 at 273, 298 & 323 K; (b) CO_2 adsorption of COP-214-MW at 273, 298 & 323 K; (c) CH_4 and N_2 uptake isotherms of COP-214 at 273 K; (d) H_2 uptake of COP-214 and COP-214-MW at 77 K. All the gas capture analyses were performed three times to show reproducibility. Error bars represent the variation.

The isosteric heats of adsorption (Q_{st}) for CO_2 were determined from CO_2 isotherms at 273, 288, and 298 K. The Q_{st} of COP-214 for CO_2 was found to be in the range of 32.9 and 33.1 kJ mol^{-1} (Figure S13c). These Q_{st} values could be attributed to the microporosity, and basic pyrazole moieties of COP-214. Although the high Q_{st} values suggest the CO_2-philic nature of polymers, the reversible CO_2 adsorption-desorption isotherms reveal a physisorption mechanism rather than chemisorption.[S13,S14]

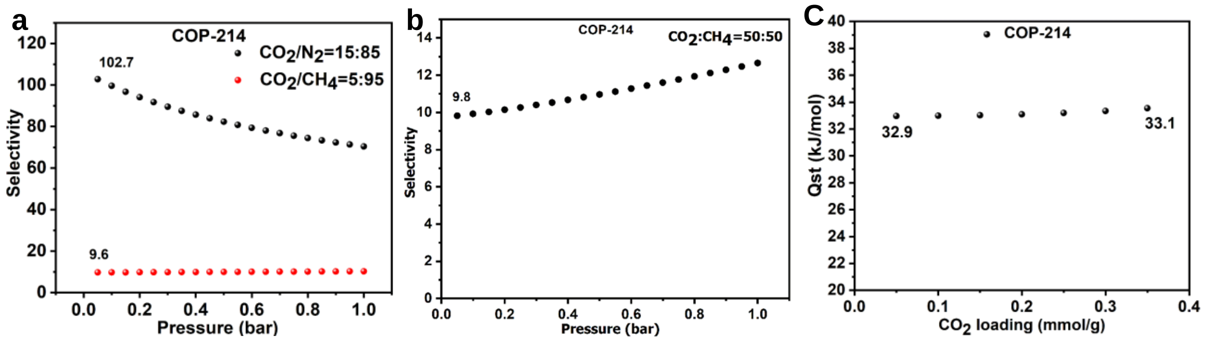

Figure S13. (a) Ideal Adsorbed Solution Theory (IAST) based gas selectivities for CO_2/N_2=15:85 and CO_2/CH_4 = 05:95 at 273 K. COP-214 shows high CO_2/N_2 selectivity of 102 and CO_2/CH_4 selectivity of 9. (c) Selectivity for CO_2/CH_4 = 50:50 at 273 K of COP-214. (c) The isosteric heat of adsorption for CO_2 derived from 273, 288, and 298 K CO_2 uptake data using the Clausius-Clapeyron equation. Q_{st} of COP-214 (32.9) shows good affinity toward the CO_2.

2.2. Metal capture study

The concentration of the metals in the samples were determined by ICP-MS analysis. The adsorption amount of each metal was calculated based on the concentrations of the control and the experimental group:

$$Adsorption\ efficiency\ (\%) = \frac{Cc-Ce}{Cc} \times 100 \quad (1)$$

Here, Cc and Ce are the average concentrations of the control group and the experimental group, respectively.

In metal selective analysis, the multi-element standard solutions (10 ppm for each metal) for ICP-MS were diluted to 100 ppb with the addition of DIW. In 10 mL of the solution, 10 mg of adsorbent (COP-214) was added for experiment.

$$\text{Metal quantity} = \frac{100\text{ug/L} \times 0.01\text{L} \times \text{adsorption efficiency}(\%)}{100} \quad (2)$$

Palladium Adsorption

The equilibrium adsorption amount of COP-214 for Pd(II) was calculated using eq.

$$Qe = \frac{\text{Co} - \text{Ce}}{\text{m}} \times V \quad (3)$$

where qe is the amount of adsorbed Pd(II) (mg g^{-1}) and C_i (mg L^{-1}) and C_e (mg L^{-1}) are the initial and equilibrium concentrations of the metal ions, respectively. V (L) refers to the

volume of the solution, and m (g) is the mass of the adsorbent. The adsorption isotherm is a representation of q_e as a function of c_e.

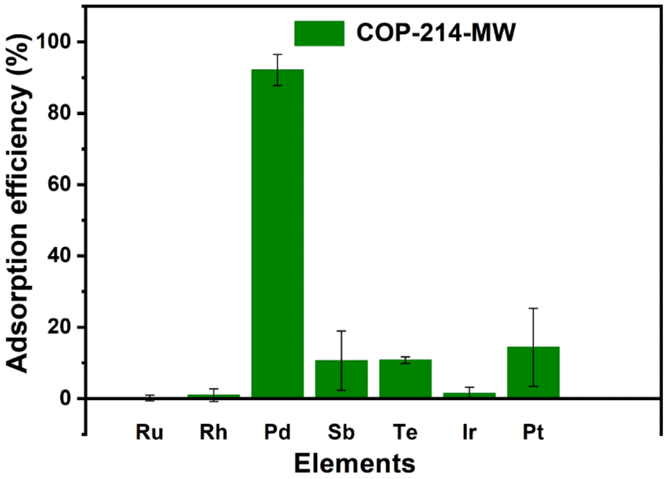

Figure S14. Precious metals adsorption selectivity of COP-214-MW. It also shows high Pd selectivity in presence of Pt. Bars correspond to standard errors of triplicate samples.

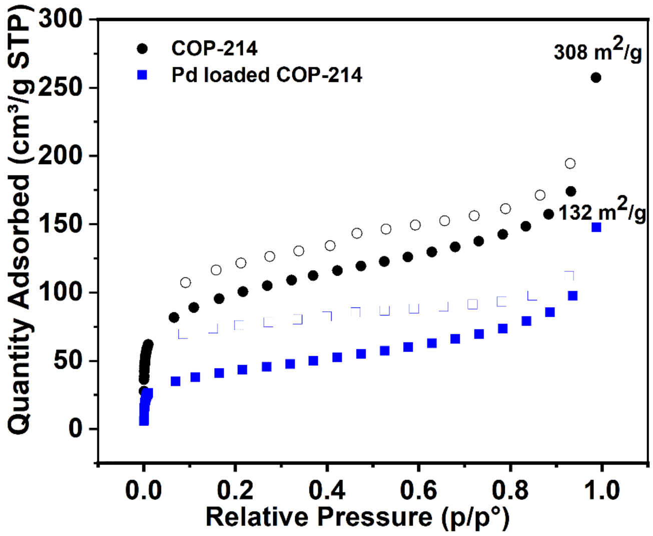

Figure S15. Ar physisorption isotherms of COP-214 and palladium loaded COP-214 at 87 K; filled and empty symbols represent adsorption and desorption, respectively. BET specific surface areas of Pd loaded COP-214 (132 m^2g^{-1}) was decreased in comparison to the parent COP-214 (308 m^2g^{-1}).

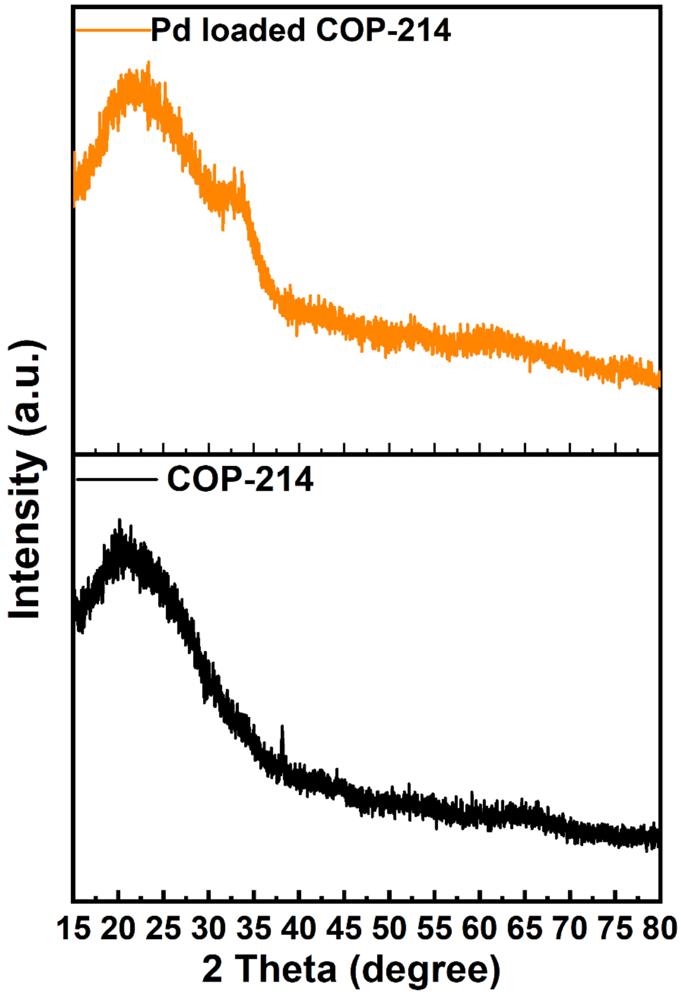

Figure S16. XRD patterns of COP-214 and palladium-loaded COP-214. The pattern of Pd captured within COP-214 does not shows any elemental palladium peaks confirming no reductive adsorption.

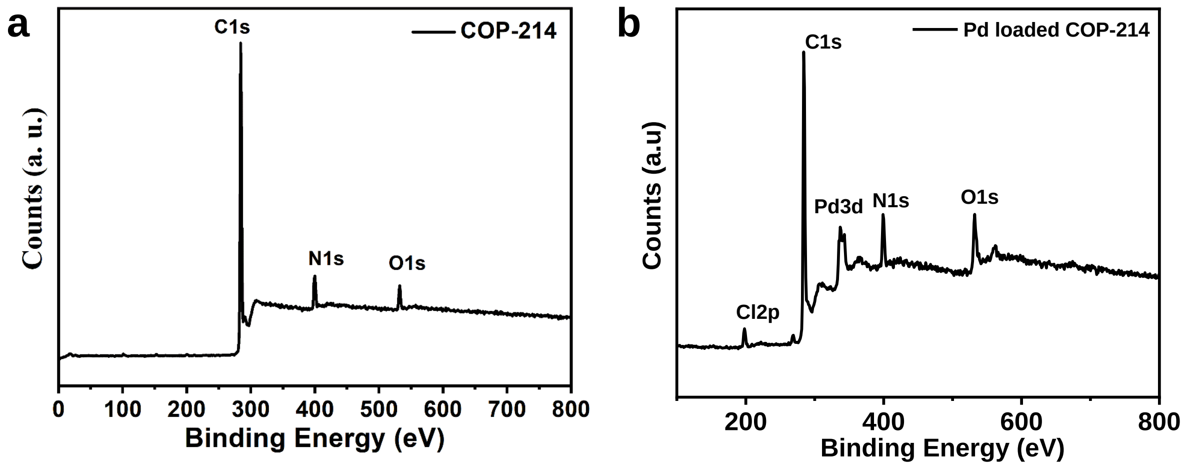

Figure S17. Overall XPS data of (a) COP-214, (b) palladium loaded COP-214. The additional peaks of Pd3d and Cl2p appear at 340.03 eV and 197.83 eV, respectively, after Pd loading in COP-214.

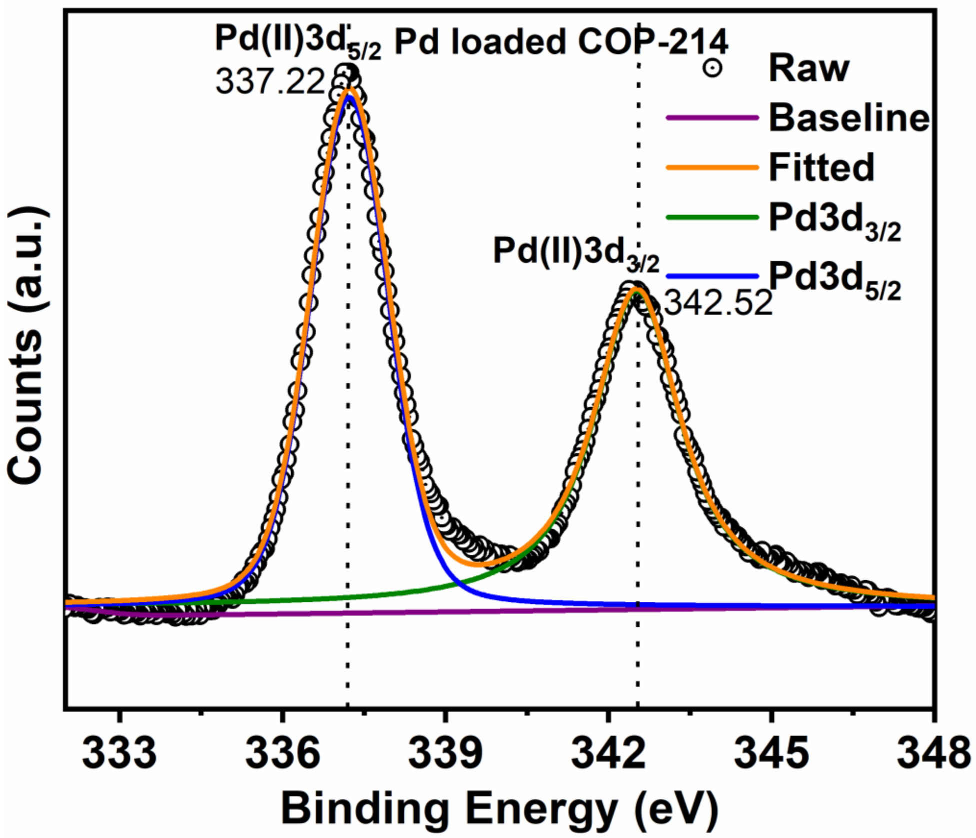

Figure S18. XPS data of Pd (3d) of palladium loaded COP-214. The two peaks are denoted Pd(II) $3d_{5/2}$ and Pd(II) $3d_{3/2}$ state. Data analysis and quantification were performed using Origin 9.60® software. A Voigt model was used in the curve fitting of the spectra.

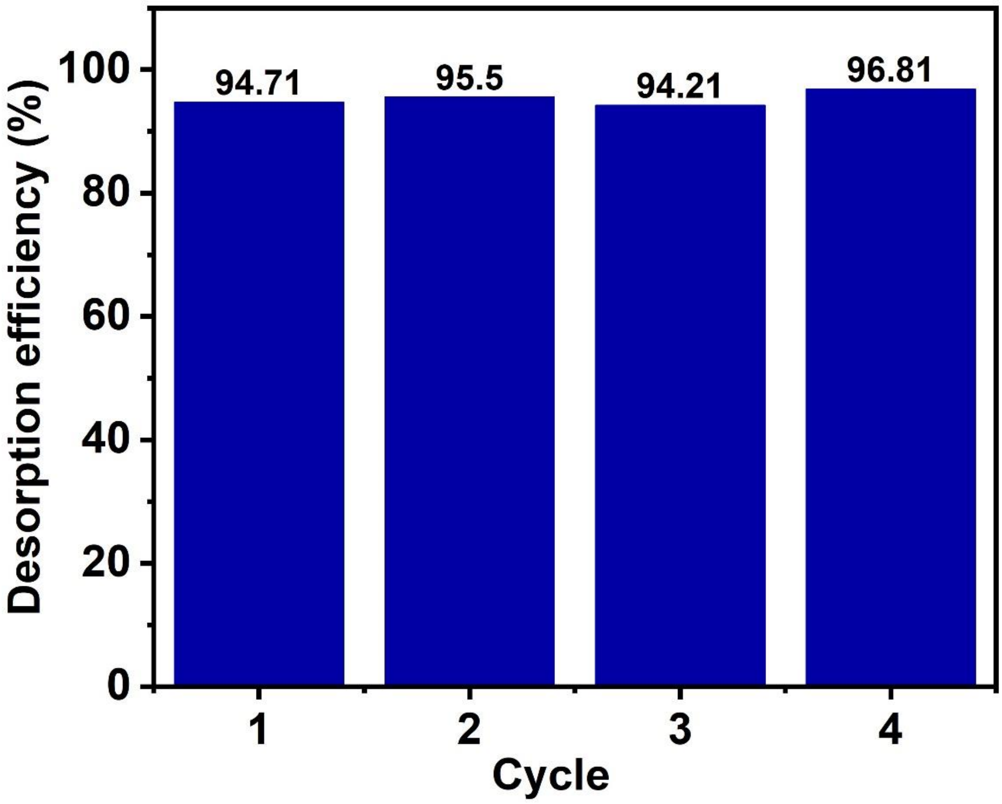

Figure S19. Palladium desorption efficiencies in 0.1 M $SC(NH_2)_2$ and 1 M HCl desorption solutions during the four repeated adsorption and desorption processes.

The adsorbent could be useful up to four ad-desorption cycles. The palladium adsorption amount was increasing at every next adsorption process due to the remaining palladium in the adsorbent after the desorption process. More than 94 % of the desorption efficiencies were maintained during the repeated uses of the adsorbent. At the fifth trial, the palladium adsorption amount was decreased to 5.90 % and the desorption efficiency was found to be 68.81 %.

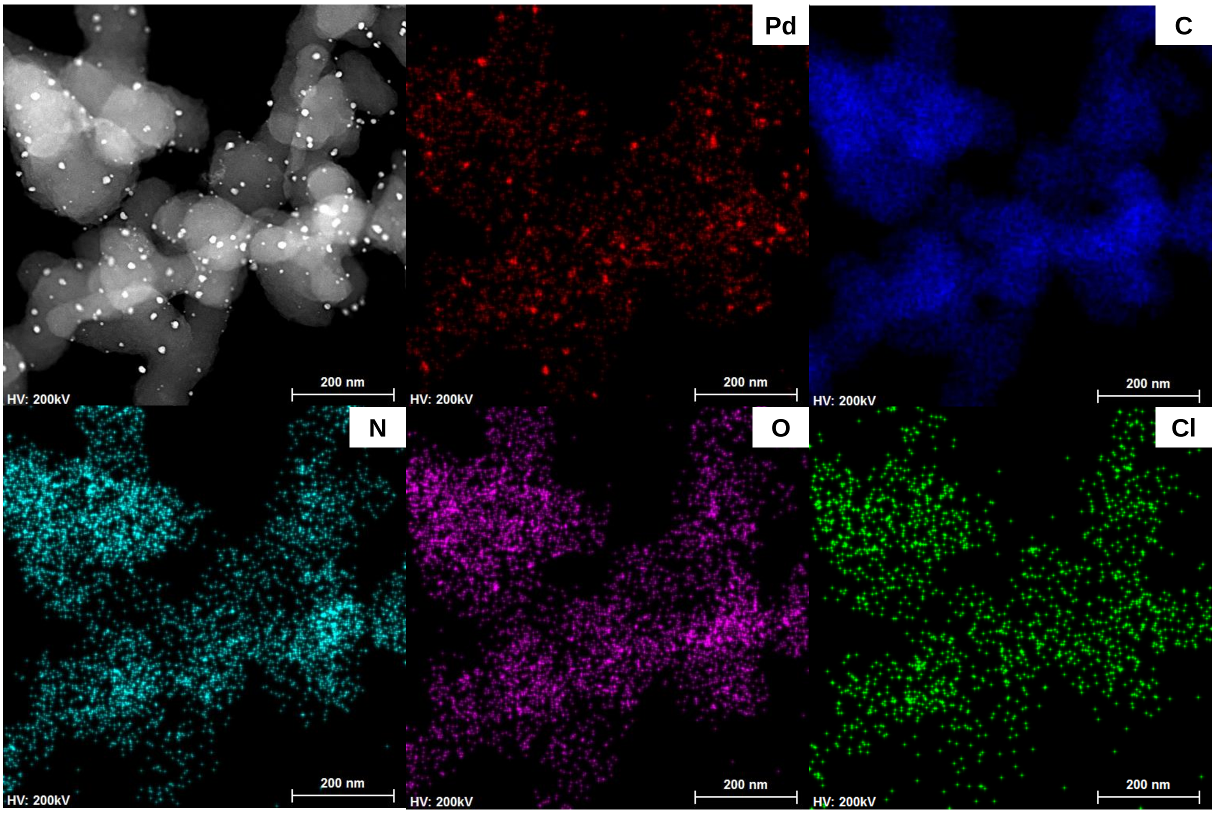

Figure S20. Transmission electron microscopy (TEM) and elemental mapping of the palladium-loaded COP-214.

3. X-ray crystallography of 6

Table S1. Crystal data and structure refinement for **6**.

Identification code	1996618	
Empirical formula	C30 H24 N4	
Formula weight	440.53	
Temperature	223(2) K	
Wavelength	0.71073 Å	
Crystal system	Monoclinic	
Space group	$P2_1/c$	
Unit cell dimensions	a = 5.7350(2) Å	α= 90°.
	b = 12.3262(4) Å	β= 97.9582(13)°.
	c = 16.2359(5) Å	γ= 90°.
Volume	1136.67(6) $Å^3$	
Z	2	
Density (calculated)	1.287 Mg/m^3	
Absorption coefficient	0.077 mm^{-1}	
F(000)	464	
Crystal size	0.270 x 0.190 x 0.130 mm^3	
Theta range for data collection	2.533 to 28.360°.	
Index ranges	-7<=h<=7, -16<=k<=16, -21<=l<=21	
Reflections collected	34672	
Independent reflections	2822 [R(int) = 0.0355]	
Completeness to theta = 25.242°	99.3 %	
Absorption correction	Semi-empirical from equivalents	
Max. and min. transmission	0.7457 and 0.7197	
Refinement method	Full-matrix least-squares on F^2	
Data / restraints / parameters	2822 / 0 / 191	
Goodness-of-fit on F^2	1.078	
Final R indices [I>2sigma(I)]	R1 = 0.0424, wR2 = 0.1090	
R indices (all data)	R1 = 0.0521, wR2 = 0.1196	
Extinction coefficient	n/a	
Largest diff. peak and hole	0.269 and -0.203 e.$Å^{-3}$	

Table S2. Atomic coordinates (x 10^4) and equivalent isotropic displacement parameters ($Å^2$x 10^3)
for **6** U(eq) is defined as one third of the trace of the orthogonalized U^{ij} tensor.

	x	y	z	U(eq)
N(1)	9283(2)	1043(1)	3254(1)	32(1)
N(2)	10663(2)	605(1)	2717(1)	37(1)
C(1)	10507(2)	1304(1)	2089(1)	38(1)
C(2)	9027(2)	2176(1)	2211(1)	38(1)
C(3)	8259(2)	1990(1)	2968(1)	31(1)
C(4)	6747(2)	2643(1)	3436(1)	31(1)
C(5)	4707(2)	3110(1)	3022(1)	39(1)
C(6)	3256(3)	3737(1)	3447(1)	45(1)
C(7)	3828(3)	3900(1)	4292(1)	44(1)
C(8)	5860(3)	3448(1)	4712(1)	41(1)
C(9)	7319(2)	2826(1)	4290(1)	36(1)
C(10)	11849(3)	1097(2)	1374(1)	55(1)
C(11)	8718(2)	378(1)	3923(1)	31(1)
C(12)	6706(2)	-227(1)	3809(1)	36(1)
C(13)	6177(2)	-927(1)	4445(1)	38(1)
C(14)	7635(2)	-986(1)	5184(1)	34(1)
C(15)	9709(2)	-347(1)	5322(1)	30(1)

Table S3. Bond lengths [Å] and angles [°] for **6**.

N(1)-C(3)	1.3597(16)
N(1)-N(2)	1.3678(14)
N(1)-C(11)	1.4330(15)
N(2)-C(1)	1.3289(17)
C(1)-C(2)	1.400(2)
C(1)-C(10)	1.5006(18)
C(2)-C(3)	1.3812(17)
C(2)-H(2)	1.003(18)
C(3)-C(4)	1.4691(17)
C(4)-C(5)	1.3911(18)
C(4)-C(9)	1.3981(17)
C(5)-C(6)	1.387(2)
C(5)-H(5)	0.981(17)
C(6)-C(7)	1.381(2)
C(6)-H(6)	0.979(17)
C(7)-C(8)	1.384(2)
C(7)-H(7)	0.990(19)
C(8)-C(9)	1.3845(19)
C(8)-H(8)	0.984(17)
C(9)-H(9)	0.952(17)
C(10)-H(10A)	0.9700
C(10)-H(10B)	0.9700
C(10)-H(10C)	0.9700
C(11)-C(12)	1.3642(18)
C(11)-C(15)#1	1.4177(17)
C(12)-C(13)	1.4105(18)
C(12)-H(12)	0.975(17)
C(13)-C(14)	1.3655(19)
C(13)-H(13)	0.998(17)
C(14)-C(15)	1.4186(18)
C(14)-H(14)	0.984(16)
C(15)-C(11)#1	1.4177(17)
C(15)-C(15)#1	1.427(2)
C(3)-N(1)-N(2)	112.44(10)
C(3)-N(1)-C(11)	128.20(10)
N(2)-N(1)-C(11)	117.95(10)

C(1)-N(2)-N(1)	104.49(11)
N(2)-C(1)-C(2)	111.51(11)
N(2)-C(1)-C(10)	119.73(13)
C(2)-C(1)-C(10)	128.76(13)
C(3)-C(2)-C(1)	105.86(11)
C(3)-C(2)-H(2)	125.1(10)
C(1)-C(2)-H(2)	128.6(10)
N(1)-C(3)-C(2)	105.70(11)
N(1)-C(3)-C(4)	123.44(10)
C(2)-C(3)-C(4)	130.81(11)
C(5)-C(4)-C(9)	118.60(12)
C(5)-C(4)-C(3)	119.59(11)
C(9)-C(4)-C(3)	121.80(11)
C(6)-C(5)-C(4)	120.84(13)
C(6)-C(5)-H(5)	121.0(10)
C(4)-C(5)-H(5)	118.1(10)
C(7)-C(6)-C(5)	119.95(14)
C(7)-C(6)-H(6)	120.6(10)
C(5)-C(6)-H(6)	119.4(10)
C(6)-C(7)-C(8)	119.93(13)
C(6)-C(7)-H(7)	120.7(11)
C(8)-C(7)-H(7)	119.4(11)
C(7)-C(8)-C(9)	120.33(13)
C(7)-C(8)-H(8)	119.5(10)
C(9)-C(8)-H(8)	120.2(10)
C(8)-C(9)-C(4)	120.34(13)
C(8)-C(9)-H(9)	119.2(10)
C(4)-C(9)-H(9)	120.4(10)
C(1)-C(10)-H(10A)	109.5
C(1)-C(10)-H(10B)	109.5
H(10A)-C(10)-H(10B)	109.5
C(1)-C(10)-H(10C)	109.5
H(10A)-C(10)-H(10C)	109.5
H(10B)-C(10)-H(10C)	109.5
C(12)-C(11)-C(15)#1	121.85(11)
C(12)-C(11)-N(1)	119.04(11)
C(15)#1-C(11)-N(1)	119.09(11)
C(11)-C(12)-C(13)	119.89(12)
C(11)-C(12)-H(12)	119.4(10)

C(13)-C(12)-H(12)	120.7(10)
C(14)-C(13)-C(12)	120.54(12)
C(14)-C(13)-H(13)	119.4(9)
C(12)-C(13)-H(13)	120.0(9)
C(13)-C(14)-C(15)	120.52(12)
C(13)-C(14)-H(14)	119.5(9)
C(15)-C(14)-H(14)	120.0(9)
C(11)#1-C(15)-C(14)	122.83(11)
C(11)#1-C(15)-C(15)#1	117.68(14)
C(14)-C(15)-C(15)#1	119.49(14)

Symmetry transformations used to generate equivalent atoms:
#1 -x+2,-y,-z+1

Table S4. Hydrogen coordinates (x 10^4) and isotropic displacement parameters (Å^{2}x 10 ^{3}) for **6**.

	x	y	z	U(eq)
H(2)	8720(30)	2853(14)	1870(11)	48(4)
H(5)	4330(30)	2986(14)	2422(11)	47(4)
H(6)	1800(30)	4035(14)	3146(10)	49(5)
H(7)	2790(30)	4335(16)	4604(11)	58(5)
H(8)	6250(30)	3564(14)	5315(11)	50(5)
H(9)	8710(30)	2517(14)	4590(10)	45(4)
H(10A)	13518	1053	1578	82
H(10B)	11570	1686	976	82
H(10C)	11327	419	1106	82
H(12)	5660(30)	-177(14)	3283(10)	48(4)
H(13)	4690(30)	-1359(13)	4366(10)	45(4)
H(14)	7240(30)	-1481(13)	5620(10)	39(4)

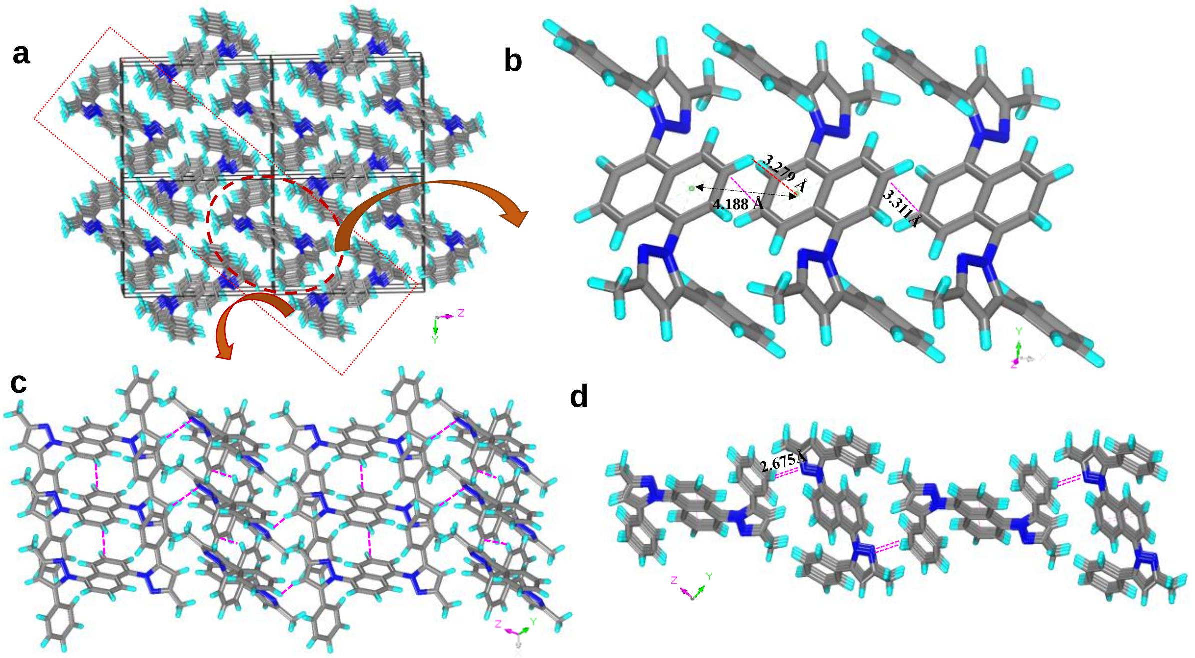

Figure S21. Illustrations for the crystal structure of **6**: (a) unit cell packing along YZ-plane. (b) C-H···π interactions of each molecule of **6** along the X- axis. Slice of the 2D-layer along YZ-plane through C-H···π and C-H···N stacking (c) side view and (d) top view.

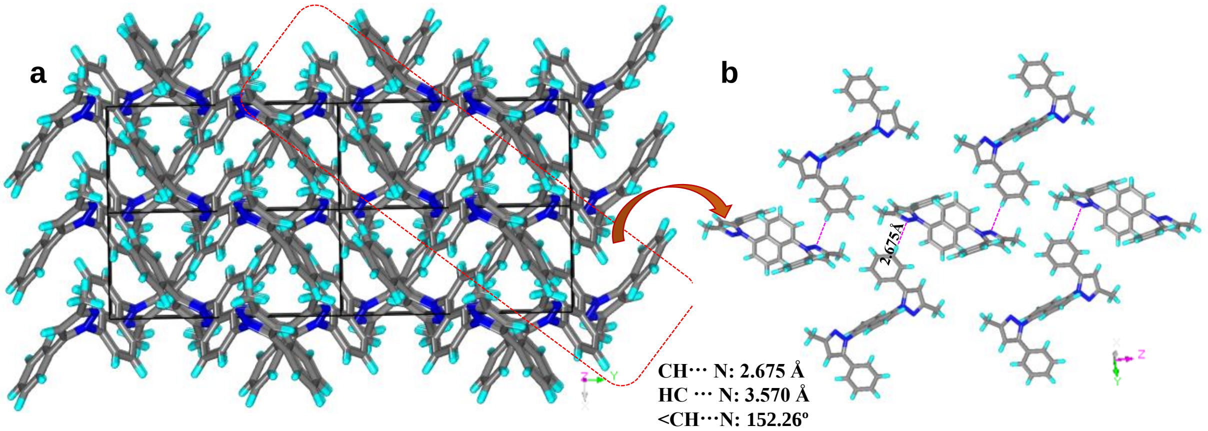

Figure S22. Illustrations for the crystal structure of **6**: (a) unit cell packing along XY-plane. (b) C-H···N stacking of each molecule of **6** along the Z- axis forming 1D chain.

Table S5. Experimental conditions for the optimization of the COP-214 synthesis (solvent, reaction conditions and surface areas (N_2 and Ar adsorption isotherms)).

	**Solvent**	**Reaction condition**	**Adsorption isotherms**	**Surface area (m^2/g)**
COP-214_dioxane+mesitylene	Dioxane+mesitylene (4:3) 6M AcOH	120°C, 72hrs (ampoule reaction)	N_2 (77K)	172.93
COP-214_ACN+DMAc+DCB	Acetonitrile+Dimethylacetamide+1,2-Dichlorobenzene (4:2:2) 6M AcOH	120°C, 72hrs (ampoule reaction)	N_2 (77K)	60.82
COP-214_ACN+DCB	Acetonitrile+1,2-Dichlorobenzene (4:3) 6M AcOH	120°C, 72hrs (ampoule reaction)	N_2 (77K)	16.70
COP-214_MeOH+THF	Methanol+Tetrahydrofuran (4:3) 6M AcOH	120°C, 72hrs (ampoule reaction)	N_2 (77K)	15.63
COP-214_^{t}BuOH+DCB	tert-Butyl alcohol+1,2-Dichlorobenzene 6M AcOH	120°C, 72hrs (ampoule reaction)	N_2 (77K)	15.73
COP-214_dioxane+mesitylene	Dioxane+mesitylene (4:3) 6M AcOH	120°C, 72hrs (ampoule reaction)	Ar (87K)	308
COP-214_dioxane+mesitylene_MW	Dioxane+mesitylene (4:3) 6M AcOH	120°C, 2hrs (Microwave)	Ar (87K)	125

4. Elemental composition of palladium adsorbed COP-214 and desorption efficiencies of COP-214

Table S6. Adsorption efficiency (%) and Metal quantity (ug) by using ICP-MS analysis.

**Metal**	**Adsorption efficiency (%)**	**Metal quantity (ug)**
Pd	92.5583	0.925583
Sb	0.1	0.001
V	9.9771	0.099771
Cr	1.6028	0.016028
Fe	6.2309	0.062309
Co	1.5818	0.015818
Cu	1.1460	0.011460
Cd	2.2539	0.022539
Tl	2.655	0.02655
Pb	11.7395	0.117395
U	2.9429	0.029429

Table S7. Elemental composition of palladium adsorbed COP-214. Metal amounts were measured by ICP-MS.

**Sample**	**C**	**N**	**H**	**O**	**Metal**
**COP-214**	74.30	6.93	4.45	7.38	
**COP-214-Pd**	72.36	7.37	4.47	3.19	5.67

Table S8. Palladium desorption conditions and efficiencies of COP-214.

No.	Desorption solution	24 h	3 h
1	0.1 M $SC(NH_2)_2$ + 1 M H_2SO_4	100	96
2	0.1 M $SC(NH_2)_2$ + 1 M HCl	100	99
3	0.1 M $SC(NH_2)_2$ + 1 M HNO_3	100	100
4	0.1 M $SC(NH_2)_2$ + 1 M HCl+ 1 M HNO_3	100	86

Table S9 Comparison of selective Pd sorption with reported adsorbents.

**No**	**Adsorbent**	**pH**	**Adsorption capacity (mg g^{-1})**		**Ref.**
			Pd (II)	Pt (IV)	
**1**	sulfur-rich MoS2 nanoflakes	0.85–2	47.6	38.5	20
**2**	Chitosan flakes	2	62.5	66.6	21
**3**	Macrocyclic ligand functionalized silica adsorbent	2	67.8	Not reported	22
**4**	Glutaraldehyde cross-linked chitosan	3	177.7	180.8	23
**5**	PU/UiO-66, PAN/UiO-66, PU/UiO-66-NH_2, and PAN/UiO-66-NH_2 (MOF-polymer fibrous menbrances)	2	146.64	87.38	24
**6**	MOF-802, UiO-66 and MOF-808	2	163.9	Not reported	25
**7**	Pyrazole based COP-214	2	103	0	This study

Section S1. Fitting parameters for the XPS data (a) N1s, before Pd uptake. (b) N1s, after Pd uptake. (c) Pd3d, after Pd uptake

(a)

Model	Gaussian	
Equation	y = y0 + A/(w*sqrt(pi/(4*ln(2)))) * exp(-4*ln(2)*(x-xc)^2/w^2)	
Plot	-N-C	-C=N
y0	15.21646 ± 34.81248	15.21646 ± 34.81248
xc	400.4762 ± 0.00726	398.70953 ± 0.00684
A	27826.89705 ± 325.20008	23811.1267 ± 304.97937
w	1.37735 ± 0.01827	1.18575 ± 0.01635
Reduced Chi-Sqr	328347.40752	
R-Square (COD)	0.9903	
Adj. R-Square	0.99013	

(b)

Model	Lorentz		
Equation	y = y0 + (2*A/pi)*(w/(4*(x-xc)^2 + w^2))		
Plot	-N-C	C=N···Pd	-N=C
y0	-374.04102 ± 19.64827	-374.04102 ± 19.64827	-374.04102 ± 19.64827
xc	398.65317 ± 0.0303	399.57184 ± 0.03862	400.41892 ± 0.03671
w	1.10085 ± 0.08213	1.25452 ± 0.2895	1.3351 ± 0.06948
A	8769.76903 ± 1450.86695	8800.11726 ± 3528.61122	14331.29887 ± 2133.95523
Reduced Chi-Sqr	67084.39804		
R-Square (COD)	0.99102		
Adj. R-Square	0.99079		

(c)

Model	Voigt	
Equation	y = nlf_voigt(x,y0,xc,A,wG,wL);	
Plot	Pd(II)3$d_{3/2}$	Pd(II)3$d_{5/2}$
y0	1012.10507 ± 110.03564	1012.10507 ± 110.03564
xc	342.52029 ± 0.00947	337.22878 ± 0.00495
A	99981.3659 ± 1879.26148	107317.91089 ± 1555.27448
wG	0.52087 ± 0.17119	1.5291 ± 0.04116
wL	1.77712 ± 0.08107	0.32235 ± 0.05694
Reduced Chi-Sqr	1676478.75017	

R-Square (COD) 0.99112

Adj. R-Square 0.99095

Section S2. Fitting parameters for COP-214 after Pd uptake.

Model	Langmuir (User)		
Equation		q=(Qsat*K*c)/(1+K*c)	
Reduced Chi-Sqr			3.69071
Adj. R-Square		0.99715	
		Value	Standard Error
C	Qsat	102.81419	4.08545
C	K	0.00519	6.96526E-4

5. Theoretical methodology

All the density functional theory (DFT) geometry optimization were performed with Gaussian 16 program package[S15]. The electronic exchange and correlation energy contribution to the total electronic energy was approximated with B3LYP[S16] hybrid functional. All intermediate and transition state geometries were optimized with 6-311G** basis set[S17] for main group atoms Pd and Pt atoms were described by Los Alamos relativistic effective core potential (ECP)[S18] and its corresponding lanl2dz basis set[S19]. The optimized geometries characterized as the local minima on the potential energy surfaces do not contain any imaginary frequency. The solvation calculations utilized a self-consistent reaction field (SCRF) approach on the gas phase geometry to model the solvation shell of dielectric constant ε = 80.1

Coordinates:

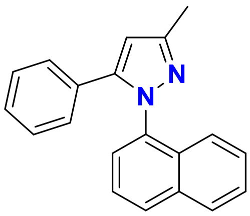

C	0.428233000	-0.694259000	1.830001000
C	0.698194000	0.106232000	0.737457000
C	1.923502000	-0.025025000	0.009126000
C	2.866959000	-1.013858000	0.453348000
C	2.554915000	-1.826822000	1.579629000
C	1.360573000	-1.673638000	2.250118000
H	1.532908000	1.518872000	-1.469394000
H	-0.510341000	-0.564085000	2.370280000

C	2.241337000	0.762281000	-1.134247000
C	4.092559000	-1.163842000	-0.256428000
H	3.280458000	-2.576632000	1.903566000
H	1.127173000	-2.297686000	3.114737000
C	4.373425000	-0.384157000	-1.358415000
C	3.436303000	0.583976000	-1.801295000
H	4.807538000	-1.914597000	0.088794000
H	5.316474000	-0.510478000	-1.894487000
H	3.664415000	1.194543000	-2.677666000
N	-0.266511000	1.083915000	0.333297000
C	-1.588036000	0.898742000	-0.013960000
C	-2.133359000	2.172692000	-0.115131000
H	-3.149958000	2.416265000	-0.412161000
N	0.045551000	2.405763000	0.463663000
C	-2.228244000	-0.404708000	-0.270008000
C	-1.570999000	-1.436674000	-0.967048000
C	-3.556689000	-0.613630000	0.147867000
C	-2.223544000	-2.643169000	-1.230505000
H	-0.550111000	-1.288506000	-1.321856000
C	-4.207445000	-1.820147000	-0.120215000
H	-4.075637000	0.173989000	0.697805000
C	-3.542709000	-2.841145000	-0.807913000
H	-1.699462000	-3.430749000	-1.776532000
H	-5.236764000	-1.964645000	0.215458000
H	-4.050367000	-3.785644000	-1.015297000
C	-1.078122000	3.069551000	0.184885000
C	-1.106967000	4.567503000	0.208699000
H	-0.125720000	4.962230000	0.507621000
H	-1.359137000	4.978890000	-0.782557000
H	-1.864064000	4.941079000	0.917180000

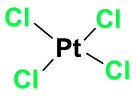

Pt	-0.000428000	0.000123000	0.000042000
Cl	-1.738079000	-1.651132000	0.000040000

Cl 1.795755000 -1.596923000 -0.000136000

Cl 1.739309000 1.648743000 0.000040000

Cl -1.795022000 1.598748000 -0.000136000

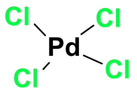

Cl-1.652912000 -1.655295000 -0.012187000

Cl 1.820891000 -1.503107000 0.022087000

Cl 1.652526000 1.656052000 -0.012313000

Cl -1.820976000 1.502343000 0.021937000

Pd 0.000174000 0.000003000 -0.007215000

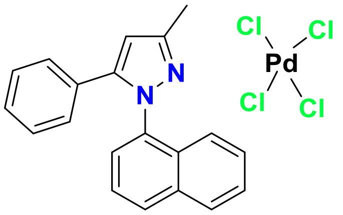

Cl -2.741957000 -2.752709000 0.225780000

Cl -4.420827000 0.146788000 0.048822000

Cl -1.888660000 1.540420000 -1.690249000

Cl-0.260434000 -1.408165000 -1.596786000

P-2.321403000 -0.600607000 -0.715975000

C 1.561745000 0.547793000 -1.109395000

C 1.664775000 -0.266124000 0.101972000

C 2.617006000 -1.363599000 0.141736000

C 3.523350000 -1.474707000 -0.967002000

C 3.458462000 -0.564559000 -2.064131000

C 2.486963000 0.420647000 -2.139903000

H 2.085072000 -2.193868000 2.063756000

H 0.737775000 1.254993000 -1.188093000

C 2.765529000 -2.256397000 1.219860000

C 4.519780000 -2.469855000 -0.956650000

H 4.182170000 -0.679593000 -2.873608000

H 2.407062000 1.063763000 -3.015296000

C 4.635273000 -3.356254000 0.117427000

C 3.763539000 -3.240432000 1.200991000

H 5.210361000 -2.533064000 -1.799083000

H	5.406463000	-4.127442000	0.108152000
H	3.847348000	-3.924107000	2.046798000
N	0.905845000	0.111615000	1.166577000
C	0.415835000	1.430212000	1.458738000
C	-0.452568000	1.262179000	2.496699000
H	-0.984620000	2.045627000	3.027740000
N	0.407411000	-0.807967000	2.041946000
C	0.957079000	2.652203000	0.861978000
C	2.350153000	2.810896000	0.666140000
C	0.090551000	3.720675000	0.555989000
C	2.853808000	4.006077000	0.148737000
H	3.041641000	2.023521000	0.969314000
C	0.604597000	4.909273000	0.044608000
H	-0.984008000	3.596332000	0.689861000
C	1.985365000	5.054130000	-0.164587000
H	3.929689000	4.120625000	0.007417000
H	-0.073434000	5.727670000	-0.203871000
H	2.379803000	5.989525000	-0.566003000
C	-0.429595000	-0.136191000	2.825614000
C	-1.210371000	-0.826042000	3.884416000
H	-2.289433000	-0.717505000	3.686623000
H	-0.954687000	-1.891717000	3.929521000
H	-1.015183000	-0.359603000	4.863934000

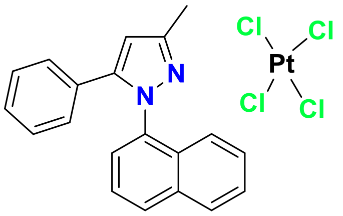

Pt	-2.294025000	-0.470948000	0.503210000
Cl	-0.555396000	0.229334000	1.911192000
Cl	-2.681376000	1.787588000	-0.157960000
Cl	-4.128189000	-1.163587000	-0.805378000
Cl	-1.916790000	-2.733994000	1.170870000
C	2.256446000	0.737347000	1.141715000
C	1.598656000	0.863118000	-0.135204000

C	1.053224000	2.145701000	-0.527402000
C	1.299405000	3.259965000	0.348241000
C	2.030790000	3.093047000	1.552490000
C	2.491459000	1.840947000	1.948619000
H	0.135013000	1.559631000	-2.393260000
H	2.558330000	-0.250351000	1.477000000
C	0.345298000	2.386080000	-1.722025000
C	0.835117000	4.546485000	-0.007173000
H	2.207972000	3.965677000	2.184061000
H	3.002667000	1.711973000	2.901993000
C	0.134958000	4.755120000	-1.195559000
C	-0.103043000	3.675526000	-2.047298000
H	1.041862000	5.382453000	0.663133000
H	-0.220279000	5.753418000	-1.453776000
H	-0.650584000	3.822915000	-2.979161000
N	1.567037000	-0.252043000	-0.940122000
C	2.489704000	-1.341051000	-0.956122000
C	1.931497000	-2.272574000	-1.785943000
H	2.373582000	-3.219814000	-2.080300000
N	0.513775000	-0.486432000	-1.776910000
C	3.823866000	-1.324407000	-0.341071000
C	4.677089000	-0.207057000	-0.478835000
C	4.292442000	-2.470764000	0.329112000
C	5.962807000	-0.235520000	0.063554000
H	4.347306000	0.668703000	-1.040221000
C	5.578657000	-2.489687000	0.867936000
H	3.636362000	-3.335357000	0.442012000
C	6.415270000	-1.372519000	0.739941000
H	6.616189000	0.630886000	-0.053491000
H	5.929399000	-3.378167000	1.396251000
H	7.421179000	-1.392497000	1.163723000
C	0.708216000	-1.702653000	-2.272831000
C	-0.270758000	-2.317351000	-3.209296000
H	-1.107616000	-1.634789000	-3.403252000

H	0.220511000	-2.570538000	-4.163180000
H	-0.660488000	-3.258192000	-2.788054000

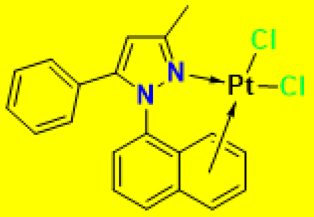

C	1.758802000	1.389698000	1.520720000
C	1.060200000	0.873305000	0.446521000
C	0.041809000	1.628545000	-0.203168000
C	-0.262020000	2.932109000	0.305239000
C	0.481141000	3.447539000	1.404780000
C	1.479582000	2.696784000	1.987329000
H	-0.247349000	0.354099000	-1.978092000
H	2.513561000	0.782612000	2.020074000
C	-0.697024000	1.121615000	-1.342629000
C	-1.297794000	3.678630000	-0.316407000
H	0.243095000	4.444538000	1.780168000
H	2.046288000	3.090555000	2.831940000
C	-2.022715000	3.179831000	-1.388841000
C	-1.702949000	1.922247000	-1.918684000
H	-1.522665000	4.673979000	0.074129000
H	-2.817659000	3.775914000	-1.838069000
H	-2.219246000	1.556206000	-2.807460000
N	1.266157000	-0.466160000	0.004145000
C	2.422614000	-1.166344000	-0.224050000
C	0.631799000	-2.496370000	-0.522747000
C	2.039381000	-2.462680000	-0.545082000
H	2.700414000	-3.277775000	-0.822740000
N	0.172014000	-1.283474000	-0.172468000
C	-0.259733000	-3.644503000	-0.865391000
H	0.322643000	-4.424835000	-1.373693000
H	-0.716581000	-4.072465000	0.038545000
H	-1.078647000	-3.321080000	-1.524155000
C	3.779767000	-0.589166000	-0.195521000

C	4.077400000	0.628173000	-0.835750000
C	4.815343000	-1.304554000	0.432470000
C	5.383612000	1.120730000	-0.837427000
H	3.290276000	1.181782000	-1.350124000
C	6.120369000	-0.806252000	0.428783000
H	4.591408000	-2.247228000	0.935317000
C	6.407621000	0.407703000	-0.203661000
H	5.603483000	2.063179000	-1.343138000
H	6.914218000	-1.367888000	0.925391000
H	7.428013000	0.796665000	-0.205901000
Cl	-3.896412000	0.493898000	0.113944000
Cl	-2.464438000	-2.245144000	1.400180000
Pt	-1.724708000	-0.478614000	-0.034397000

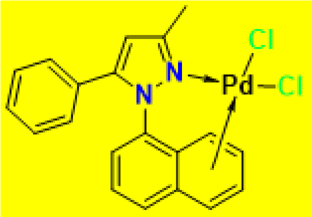

C	1.444319000	1.300094000	1.580830000
C	0.784636000	0.803650000	0.472495000
C	-0.222868000	1.563092000	-0.191208000
C	-0.557244000	2.850310000	0.341508000
C	0.146599000	3.344815000	1.474889000
C	1.135101000	2.590347000	2.071243000
H	-0.506462000	0.264628000	-1.947472000
H	2.192183000	0.688316000	2.085226000
C	-0.920702000	1.087327000	-1.360252000
C	-1.584537000	3.603891000	-0.289930000
H	-0.112864000	4.330050000	1.867036000
H	1.669936000	2.969663000	2.942947000
C	-2.266617000	3.123443000	-1.394688000
C	-1.920954000	1.874611000	-1.941209000
H	-1.833663000	4.585001000	0.121023000
H	-3.054693000	3.720256000	-1.855473000
H	-2.417077000	1.518415000	-2.845171000

N	1.034416000	-0.520192000	0.004559000
C	2.213528000	-1.174666000	-0.244681000
C	0.467505000	-2.563869000	-0.532902000
C	1.873088000	-2.481255000	-0.576727000
H	2.559514000	-3.270976000	-0.866397000
N	-0.028763000	-1.373678000	-0.161680000
C	-0.399101000	-3.729065000	-0.884390000
H	0.207528000	-4.642418000	-0.947425000
H	-1.193542000	-3.866078000	-0.138482000
H	-0.880904000	-3.568947000	-1.862946000
C	3.550717000	-0.553101000	-0.214200000
C	3.800274000	0.696522000	-0.811129000
C	4.618382000	-1.257521000	0.371573000
C	5.090164000	1.230695000	-0.812122000
H	2.988886000	1.243688000	-1.293534000
C	5.906810000	-0.718168000	0.368196000
H	4.432508000	-2.225118000	0.841527000
C	6.145997000	0.527507000	-0.221203000
H	5.272201000	2.198358000	-1.284125000
H	6.725419000	-1.272776000	0.831409000
H	7.153531000	0.948643000	-0.223250000
Pd	-1.973289000	-0.621747000	-0.058629000
Cl	-4.163319000	0.247156000	-0.042212000
Cl	-2.619114000	-2.275109000	1.520760000

6. References

[S1] T. Geiger, H. Benmansour, B. Fan,R. Hany, F. Nüesch, *Macromol. Rapid Commun.* **2008**, *29*, 651.

[S2] G. Goörlitz, H. Hartmann, Heteroatom Chemistry **1997**, *8*, 147.

[S3] M. Garai, V. Rozyyev, Z. Ullah, A. Jamal, C. T. Yavuz, APL Materials **2019**, *7*, 111102.

[S4] Bruker AXS Inc. SMART, SAINT-Plus v 6.22 and XPREP; Bruker AXS Inc.: Madison, WI, USA, **2000**.

[S5] Sheldrick, G.M. SADABS *v* 2.03; University of Göttingen: Göttingen, Germany, **2002**.

[S6] Bruker AXS Inc. SHELXTL *v* 6.10; Bruker AXS Inc.: Madison, WI, USA, **2000**.

[S7] N. A. Dogan, E. Ozdemir, C. T. Yavuz, *ChemSusChem* **2017**, *10*, 2130.

[S8] T. S. Nguyen, C. T. Yavuz, *Chem. Commun.*, **2020**, *56*, 4273.

[S9] J. X. Jiang, F. Su, A. Trewin, C. D. Wood, N. L. Campbell, H. Niu, C. Dickinson, A. Y. Ganin, M. J. Rosseinsky, Y. Z. Khimyak, A. I. Cooper, *Angew. Chem. Int. Ed.* **2007**, *46*, 8574.

[S10] O. Buyukcakir, S. H. Je, J. Park, H. A. Patel, Y. Jung, C. T. Yavuz, A. Coskun, *Chem. Eur. J.* **2015**, *21*, 15320.

[S11] Y. Jin, Z. Lei, P. Taynton, S. Huang, W. Zhang, *Matter* **2019**, *1*, 1456.

[S12] A. Kumar, X. Yang, Q. Xu, *J. Mater. Chem. A.* **2019**, *7*, 112.

[S13] N. A. Dogan, Y. Hong, E. Ozdemir, C. T. Yavuz, *ACS Sustain. Chem. Eng.* **2019**, *7*, 123.

[S14] H. A. Patel, J. Byun, C. T. Yavuz, *ChemSusChem* **2017**, *10*, 1303.

[S15] M. J. Frisch, G. W Trucks, H. B Schlegel, G. E Scuseria, M. A Robb, J. R Cheeseman, G. Scalmani, V. Barone, G. A Petersson, H Nakatsuji, 'Gaussian 16 Revision A. 03. 2**016**; Gaussian Inc., *Wallingford CT,* 2 (**2016**).

[S16] I. Y. Zhang, J. Wu, X. Xu, *Chem. Commun.*, **2010**, *46*, 3057.

[S17] C. M. Rohlfing, and P J. Hay, *J. Chem. Phys*. **1985**, *83*, 4641.

[S18] C. E. Check, T. O. Faust, J. M. Bailey, B. J. Wright, T. M. Gilbert, L. S. Sunderlin, *J.Phys. Chem. A* **2001**, *105,* 8111.

[S19] J. K. Park, B. G. Kim, I. S. Koo, *Bull. Korean Chem. Soc*. **2005**, *26,*1795.

[S20] B. Feng, C. Yao, S. Chen, R. Luo, S. Liu, S. Tong, *Chem. Eng. J*. **2018**, *350*, 692.

[S21] H. Sharififard, M. Soleimani and F. Z. Ashtiani, *Int. J. Global Warming*, **2014**, *6*, 303.

[S22] F. Bai, G. Ye, G. Chen, J. Wei, J. Wang, J. Chen, *Sep. Purif. Technol*. **2013**, *106*, 38.

[S23] S. Mincke, T. G. Asere, I. Verheye, K. Folens, F. V. Bussche, L. Lapeire, K. Verbeken, P. V. D. Voort, D. A. Tessema, F. Fufa, G. D. Laing, C. V. Stevens, *Green Chem.*, **2019**, *21*, 2295

[S24] Y. Liu, S. Lin, Y. Liu, A. K. Sarkar, J. K. Bediako, H. Yong Kim, Y.-S. Yun, *Small* **2019**, *15*, 1805242

[S25] S. Lin, Y. Zhao, J. K. Bediako, C. Cho, A. K. Sarkar, C. Lim, Y. Yun, *Chem. Eng. J*. **2019**, *362*, 280.
